# Temporal trends in frequency, type and severity of myopia and associations with key environmental risk factors in the UK: Findings from the UK Biobank Study

**DOI:** 10.1371/journal.pone.0260993

**Published:** 2022-01-19

**Authors:** Phillippa M. Cumberland, Vasiliki Bountziouka, Christopher J. Hammond, Pirro G. Hysi, Jugnoo S. Rahi, on behalf of the UK Biobank Eye and Vision Consortium

**Affiliations:** 1 Population, Policy and Practice Research and Teaching Department, UCL Great Ormond Street Institute of Child Health, London, United Kingdom; 2 Ulverscroft Vision Research Group, Ulverscroft Foundation, Leicester, United Kingdom; 3 Department of Ophthalmology, King’s College London, St Thomas’ Hospital Campus, London, United Kingdom; 4 Department of Twin Research and Genetic Epidemiology, King’s College London, St Thomas’ Hospital Campus, London, United Kingdom; 5 National Institute for Health Research (NIHR) Biomedical Research Centre, Moorfields Eye Hospital NHS Foundation Trust and UCL Institute of Ophthalmology, London, United Kingdom; National Yang-Ming University Hospital, TAIWAN

## Abstract

This study investigated temporal trends in the epidemiology of primary myopia and associations with key environmental risk factors in a UK population. Data were collected at recruitment (non-cycloplegic autorefraction, year of birth, sex, ethnicity, highest educational attainment, reason and age of first wearing glasses and history of eye disease) from 107,442 UK Biobank study participants aged 40 to 69 years, born between 1939 and 1970. Myopia was defined as mean spherical equivalent (MSE) ≤-1 dioptre (D). Temporal changes in myopia frequency by birth cohort (5-year bands using date of birth) and associations with environmental factors were analysed, distinguishing both type (childhood-onset, <18 years versus adult-onset) and severity (three categories: low -1.00 to -2.99D, moderate -3.00 to -5.99D or high ≥-6.00D). Overall myopia frequency increased from 20.0% in the oldest cohort (births 1939–1944) to 29.2% in the youngest (1965–1970), reflecting a relatively higher increase in frequency of adult-onset and low myopia. Childhood-onset myopia peaked in participants born in 1950–54, adult-onset myopia peaked in the cohort born a decade later. The distribution of MSE only shifted for childhood-onset myopia (median: -3.8 [IQR -2.4, -5.4] to -4.4 [IQR -3.0, -6.2]). The magnitude of the association between higher educational attainment (proxy for educational intensity) and myopia overall increased over time (adjusted Odds Ratio (OR) 2.7 [2.5, 2.9] in the oldest versus 4.2 [3.3, 5.2] in the youngest cohort), being substantially greater for childhood-onset myopia (OR 3.3 [2.8, 4.0] to 8.0 [4.2, 13]). Without delineating childhood-onset from adult-onset myopia, important temporal trends would have been obscured. The differential impact of educational experience/intensity on both childhood-onset and high myopia, amplified over time, suggests a cohort effect in gene-environment interaction with potential for increasing myopia frequency if increasing childhood educational intensity is unchecked. However, historical plateauing of myopia frequency does suggest some potential for effective intervention.

## Introduction

Myopia is one form of refractive error, placed at the opposite end of the distribution of this quantitative trait to hypermetropia. As it arises as a consequence of ocular growth [1] that is unchecked by normal homeostatic control, it has long intrigued clinicians and scientists. However, it is now a pressing public health concern internationally, with an emerging ‘epidemic’ of myopia, characterised by increased prevalence accompanied by a whole population shift in distribution towards younger age at onset and greater severity [2, 3]. This places a growing population of people at risk of the potentially blinding sequelae associated with greater severity of myopia, as evidenced in Asia [4, 5] but less strikingly in Europe [6–9]. The economic impact of myopia *per se* and associated visual impairment is already considerable [10] and set to escalate [11].

Severity and timing of onset of myopia (childhood versus adult-onset) are related [12]: childhood-onset myopia generally has a clear familial/hereditary basis, is progressive into adulthood and often of greater severity [13]. Onset, conventionally defined by age at first wearing optical correction, is infrequently considered in myopia research, although it offers the opportunity to investigate the relative importance of genetic predisposition and the nature of environmental risk factors: for example, to advance the current focus in both aetiological research and in preventive public health interventions, on the role of educational experience and intensity in childhood. Although severity (as a quantitative trait) is always measured in research, it is usual for this to be the intermediate rather than final measure, as the latter which can only be assigned in middle/late adulthood, and this risks non-random/systematic misclassification of severity. We hypothesised that if changing environmental factors, in particular educational experience, are accounting for increasing frequency of myopia in the UK, a cohort effect would be discernible in changing associations with myopia, with different profiles for childhood and adult-onset forms. We investigated this using the UK Biobank Study, a unique large contemporary adult population sample whose members, born over a period of more than three decades, have undergone a detailed ophthalmic examination. This affords the opportunity to analyse ‘historical’ cohorts covering a period of important socio-demographic, economic, and educational change in the UK from which current and emerging trends may be identified and examined. Drawing on our proof-of-concept study [14], we investigated whether there were differences between childhood-onset versus adult-onset myopia in temporal trends in both frequency and severity and in associations with key environmental factors.

## Methods

### Study population

Between 2006 and 2010, more than half a million UK adults aged between 40 and 69 years and registered with the UK National Health Service [15, 16] consented to participate in the UK Biobank study (UKBB). Since that time they have reported through serial questionnaires and in repeated examinations on their lifestyle, environment and medical history [16, 17]. Specifically, data have been collected on age at first use and reason for optical correction (glasses or contact lenses) and any eye illness or eye surgery. An enhanced ophthalmic assessment including non-cycloplegic autorefraction (Tomey RC 5000 auto-refkeratometer, Tomey Corp., Japan) as per the UK Biobank protocol (see participation flowchart; S1 Fig) was undertaken at the time of registration on a large subsample, as detailed elsewhere [18].

### Available UK Biobank data

Participants reported diverse socio-demographic information including: sex, age at recruitment, ethnicity using the UK Office for National Statistics classification of White, mixed, Asian or Asian British (including Indian, Pakistani and Bangladeshi), Black or Black British), Chinese, Mixed and other group, and socio-economic status assessed using conventional individual level markers (versus less granular area-based proxy markers) of housing tenure (council rental, private rental, home-ownership with a mortgage and outright ownership) available for all participants. In the absence of specific data on educational experience or intensity over the participants lifetime, we used *a priori* their highest educational attainment i.e. ‘none’, ‘O’ levels (examinations at statutory school-leaving age in the UK), ‘A’ levels (examinations at age 18 years in the UK) or higher level of education (e.g. Degree) in two ways. We used ‘no educational qualification’ a sensitive proxy for lowest educational intensity and experience during school years and the gradient of qualifications achieved from ‘O’ levels to Degree/higher Level qualification as a proxy for intensity during school years. In addition, participants reported any eye conditions, eye surgery or other treatment received including optical correction and reason for prescription, as well as age of diagnosis or treatment e.g. surgery or first prescription for glasses.

### Ethics statement

UK Biobank has received approval from the Northwest Multi-Centre Research Ethics committee, which covers the United Kingdom. UK Biobank also obtained approval in England and Wales from the National Information Governance Board for Health & Social Care, which allows access to information for inviting individuals to participate. In Scotland, UK Biobank received approval from the Community Health Index Advisory Group. Participants in the UK Biobank were voluntarily enrolled and gave their written informed consent. This research has been conducted using the UK Biobank Resource under Application Number 669.

### Outcome measures

As detailed previously [18] the conventional metric of spherical equivalent (SE) (algebraic sum of sphere + 0.5 cylinder) in diopters (D) was used to categorise severity of refractive error in a clinically meaningful way, in keeping with international guidelines [19, 20]: emmetropia (SE -0.99D to +0.99D), low myopia (SE -1.0D to -2.99D), moderate myopia (SE -3.0D to -5.99D), high myopia (SE -6.0D or more extreme), low hypermetropia (SE +1.0D to +2.99D) and moderate/high hypermetropia (SE +3.0D or more extreme) [19]. Mean spherical equivalent (MSE) of the two eyes for each individual (or single eye measure if both measurements were not available) was used to report frequency. Importantly, in order to reliably analyse primary myopia, individuals who reported treatments that might alter refraction, including cataract surgery, refractive laser surgery, vitrectomy or retinal detachment, and those with high inter-ocular discordance (hypermetropia in one eye and myopia in the other) were excluded as detailed elsewhere [18].

Individuals were also characterised as having either childhood-onset (by the age of 17 years) or adult-onset myopia using self-reported age at first glasses/contact lenses wear and reason for first optical correction, based on prior work evaluating the utility of self-report in this population [21]. To enable a ‘deep dive’, we dichotomised those in the childhood-onset group into early (under 10 years) and late (10 to 17 years) childhood onset using the conventional threshold of 18 years for defining adulthood.

Participants were assigned into one of six cohorts (five-year age bands) based on their year of birth: the oldest cohort comprising those born between 1939 and 1944 (cohort 1) and the youngest (cohort 6), births between 1965 and 1970.

### Statistical methods

Frequency and distribution of refractive error (myopia, emmetropia and hypermetropia) in the UK Biobank population, by demographic and environmental factors are reported. Multivariable logistic regression was used to model associations between myopia (all and by onset) and socio-demographic factors described earlier, including sex and ethnicity, with emmetropia as the reference category. Sandwich variance estimates allowed for correlation within test centres. Results are given as point estimates alongside interquartile range (IQR) or 95% confidence intervals. All analyses were carried out using Stata 15.0 (StataCorp, College Station, Texas).

## Results

### Participation and study sample

The final study sample of 107,442 (of 115,785 subjects eligible for ophthalmic examination, S1 Fig) was older and more affluent and with fewer males, but similar ethnic distribution as the general UK population, as detailed elsewhere [18].

### Trends in frequency of myopia

The overall frequency of primary myopia in the UK Biobank population was 26.9% [26.6%, 27.1%], comprising 4.0% [3.9%, 4.1%] high myopia, 9.5% [9.3%, 9.7%] moderate and 13.3% [13.0%, 13.6%] low myopia. Altogether 45.6% [45.3%, 45.9%] of subjects were emmetropic and 27.6% [27.3%, 27.8%] hypermetropic.

**Fig 1:**
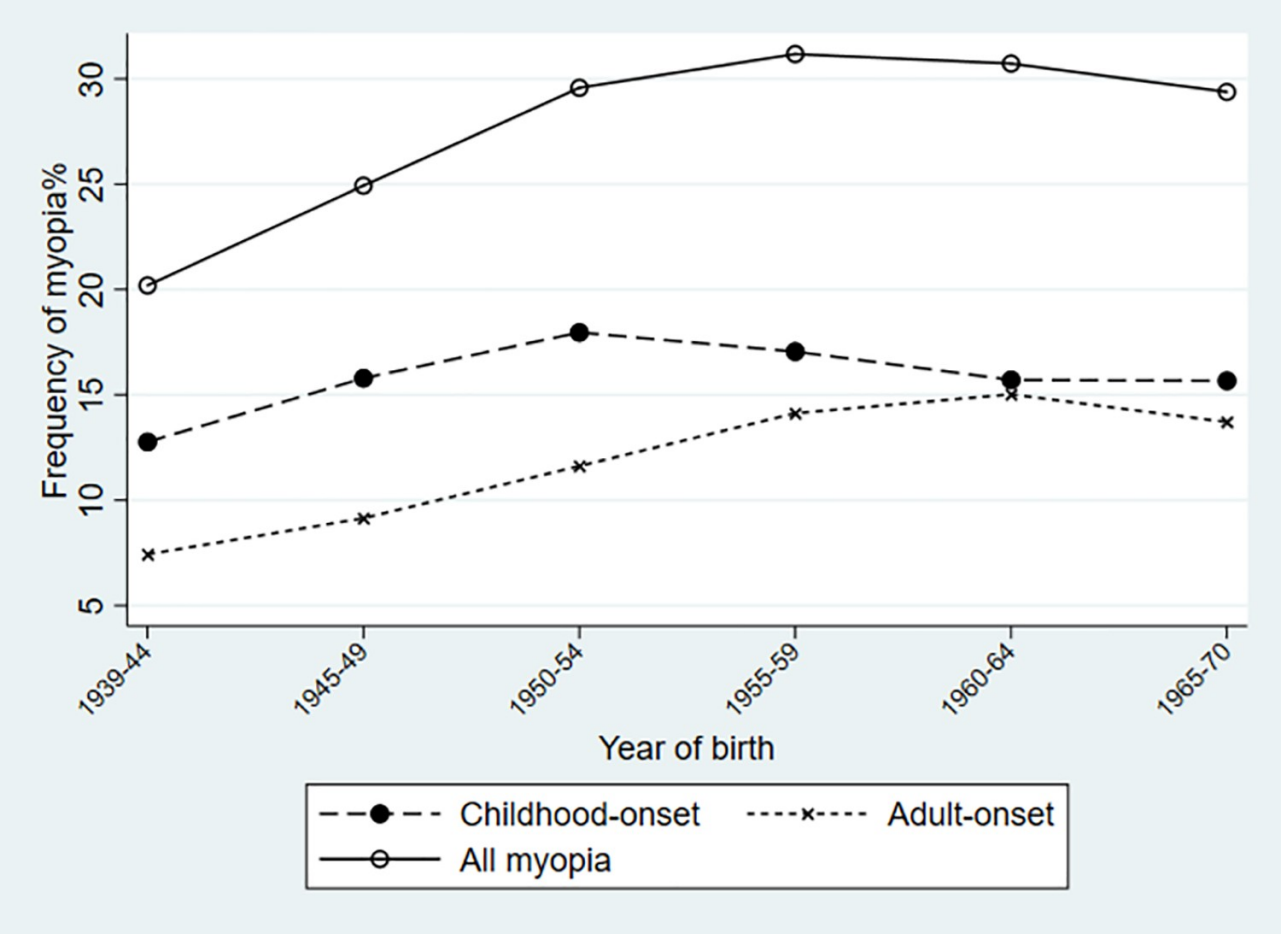
Frequency of myopia (all, childhood-onset and adult-onset). By year of birth as 5-year bands.

Fig 1 and Table 1 show that from a baseline overall myopia frequency of 20.0% (19.5% to 20.6%) amongst those born in cohort 1 (1939–44), there was a steady increase in subsequent birth cohorts, peaking in cohort 4 (1955–54) and then plateauing. However, the frequency of childhood-onset myopia peaked at 17.8% in cohort 3, two decades earlier than the peak at 15.0% of adult-onset myopia in cohort 5 (1960–1964).

**Table 1:** Frequency of myopia (all, childhood-onset and adult-onset) and emmetropia in the UK Biobank population: Distribution of socio-demographic and environmental factors.

	Overall	Myopia						Emmetropia		Hypermetropia	
Factor		Childhood-onset		Adult-onset		All myopia					
	N	n	% (95% CI)	n	% (95% CI)	n	% (95% CI)	n	% (95% CI)	n	% (95% CI)
Population	107,442	16,786	15.6 (15.4,15.8)	12,066	11.2 (11.0,11.4)	28,852	26.9 (26.6,27.1)	48,960	45.6 (45.3,45.9)	29,630	27.6 (27.3,27.8)
Year of birth											
1939–44	20,042	2,529	12.6 (12.2,13.1)	1,482	7.4 (7.0,7.8)	4,011	20.0 (19.5,20.6)	6,884	34.4 (33.7,35.0)	9,147	45.6 (45.0,46.3)
1945–49	26,963	4,219	15.6 (15.2,16.1)	2,473	9.2 (8.8,9.5)	6,692	24.8 (24.3,25.3)	10,353	38.4 (37.8,39.0)	9,918	36.8 (36.2,37.4)
1950–54	18,998	3,378	17.8 (17.2,18.3)	2,207	11.6 (11.2,12.1)	5,585	29.4 (28.8,30.0)	8,418	44.3 (43.6,45.0)	4,995	26.3 (25.7,26.9)
1955–59	16,139	2,722	16.9 (16.3,17.5)	2,263	14.0 (13.5,14.6)	4,985	30.9 (30.2,31.6)	8,081	50.1 (49.3,50.8)	3,073	19.0 (18.4,19.7)
1960–64	14,010	2,182	15.6 (15.0,16.2)	2,104	15.0 (14.4,15.6)	4,286	30.6 (29.8,31.4)	8,071	57.6 (56.8,58.4)	1,653	11.8 (11.3,12.3)
1965–70	11,290	1,756	15.6 (14.9,16.2)	1,537	13.6 (13.0,14.3)	3,293	29.2 (28.3,30 0)	7,153	63.4 (62.5,64.2)	844	7.5 (7.0,8.0)
Sex											
Female	58,445	9,689	16.6 (16.3,16.9)	6,048	10.3 (10.1,10.6)	15,737	26.9 (26.6,27.3)	25,823	44.2 (43.8,44.6)	16,885	28.9 (28.5,29.3)
Male	48,997	7,097	14.5 (14.2,14.8)	6,018	12.3 (12.0,12.6)	13,115	26.8 (26.4,27.2)	23,137	47.2 (46.8,47.7)	12,745	26.0 (25.6,26.4)
Highest educational qualification¥											
None	15,776	1,009	6.4 (6.0,6.8)	1,054	6.7 (6.3,7.1)	2,063	13.1 (12.6,13.6)	6,951	44.1 (43.3,44.8)	6762	42.9 (42.1,43.6)
O-level	28,272	3,500	12.4 (12.0,12.8)	3,344	11.8 (11.5,12 2)	6,844	24.2 (23.7,24.7)	13,849	49.0 (48.4,49.6)	7,579	26.8 (26.3,27.3)
A-level	19,161	2,854	14.9 (14.4,15.4)	2,248	11.7 (11.3,12.2)	5,102	26.6 (26.0,27.3)	8,974	46.8 (46.1,47.5)	5,085	26.5 (25.9,27.2)
Higher-level	42,767	9,329	21.8 (21.4,22.2)	5,292	12.4 (12.1,12.7)	14,621	34.2 (33.7,34.6)	18,446	43.1 (42.7,43.6)	9,700	22.7 (22.3,23.1)
*Missing*	*1*,*466*	*94*	*-*	*128*	*-*	*222*	*-*	*740*	*-*	*504*	*-*
Accommodation tenure											
Rent from council	7,299	782	10.7 (10.0,11.4)	642	8.8 (8.2,9.5)	1,424	19.5 (18.6,20.4)	3,720	51.0 (49.8,52.1)	2,155	29.5 (28.5,30.6)
Rent from private	4,351	571	13.1 (12.1,14.2)	455	10.5 (9.6,11.4)	1,026	23.6 (22.3,24.9)	2,328	53.5 (52.0,55.0)	997	22.9 (21.7,24.2)
Own with mortgage	37,853	5,937	15.7 (15.3,16.1)	5,044	13.3 (13.0,13.7)	10,981	29.0 (28.6,29.5)	19,441	51.4 (50.9,51.9)	7,431	19.6 (19.2,20.0)
Own outright	55,837	9,251	16.6 (16.3,16.9)	5,684	10.2 (9.9,10.4)	14,935	26.4 (26.4,27.1)	22,458	40.2 (39.8,40.6)	18,444	33.0 (32.6,33.4)
*Missing*	*2*,*102*	*245*	*-*	*241*	*-*	*486*	*-*	*1*,*013*	*-*	*603*	*-*
Ethnicity											
White	95,783	15,257	15.9 (15.7,16.2)	10,659	11.1 (10.9,11.3)	25,916	27.6 (26.8,27.3)	42,625	44.5 (44.2,44.8)	27,242	28.4 (28.2,28.7)
Mixed ethnicity	974	168	17.2 (15.0,19.8)	122	12.5 (10.6,14.8)	290	29.8 (27.0,32.7)	516	53.0 (49.8,56.1)	168	17.2 (15.0,19.8)
Asian or Asian British§	4,032	528	13.1 (12.1,14.2)	472	11.7 (10.7,12.7)	1,000	24.8 (23.5,26.2)	2,185	54.2 (52.6,55.7)	847	21.0 (18.5,21.0)
Black or Black British	3,758	396	10.5 (9.6,11.6)	479	12.8 (11.7,13.9)	875	23.3 (22.0,24.7)	2,143	57.0 (55.4,58.6)	740	19.7 (18.5,21.0)
Chinese	490	169	34.5 (30.3,38.8)	63	12.7 (10.1,16.1)	232	47.4 (42.9,51.8)	205	41.8 (37.5,46.3)	53	10.8 (8.3,13.9)
Other	1,627	166	10.2 (8.8,11.8)	189	11.6 (10.2,13.3)	355	21.8 (19.9,23.9)	908	55.8 (53.4,58.2)	364	22.4 (20.4,24.5)
*Missing*	*778*	*102*	*-*	*82*	*-*	*184*	*-*	*378*	*-*	*216*	*-*

¥O level: State examination at age 16 years; A level: State examination at age 18 years.

§Asian category includes Indian, Pakistani and Bangladeshi.

**Fig 2:**
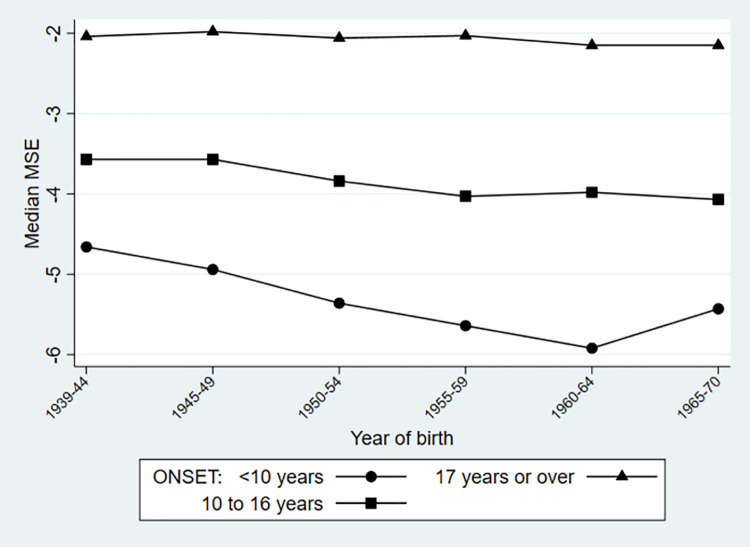
Median MSE (mean spherical equivalent): Childhood-onset myopia (subdivided: Before age 10 and aged 10 to 16 years) and adult-onset myopia, by year of birth as 5-year bands.

**Fig 3:**
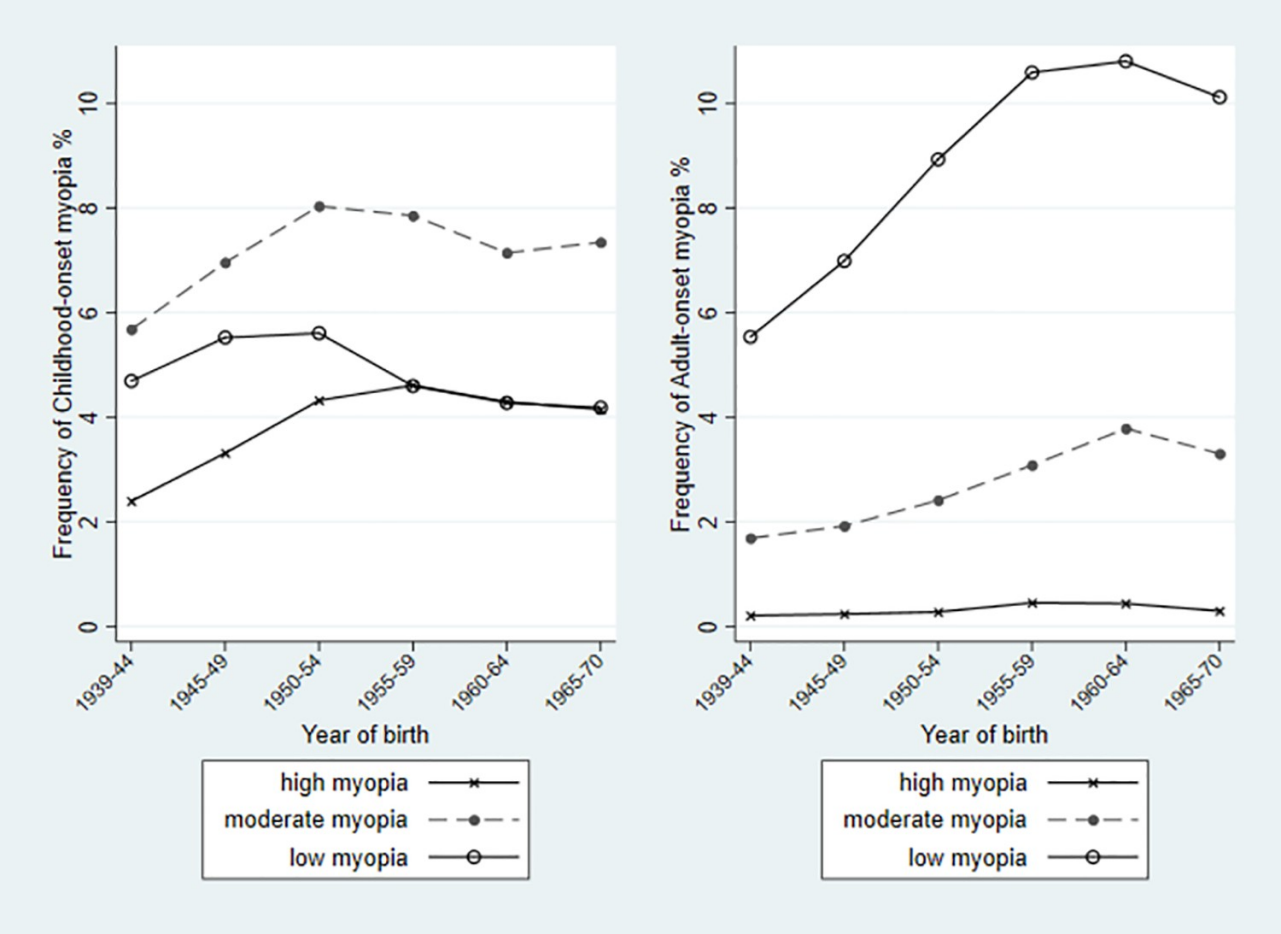
Frequency of childhood-onset and adult-onset myopia by severity (high, moderate and low), by year of birth as 5-year bands.

The overall median MSE in childhood-onset myopia decreased from -3.8D [IQR -2.4D, -5.4D] by a clinically meaningful amount of -0.6D, to -4.4D [IQR -3.0D, -6.2D] (S1 Table). Although moderate myopia accounted for more than half of all childhood-onset myopia in all cohorts, the largest relative increase over time (doubling of frequency) in childhood-onset was of high myopia, driving this change in MSE. The subgroup analysis showed that amongst those with childhood onset before the age of 10 years, the median MSE changed by -1.3D over time (cohort 1 versus cohort 5) whilst amongst those with onset between 11 and 17 years, the median MSE changed by only -0.5D (cohort 1 versus cohort 6, Fig 2; S1 Table). By contrast, the median MSE in adult-onset myopia was stable at around -2.1D. Low myopia accounted for at least three-quarters of adult-onset myopia in all birth cohorts and also had the greatest relative increase (doubling) in frequency, without any change in the small contribution of high myopia (Fig 3).

### Distribution of socio-demographic and environmental risk factors

The distribution of sociodemographic factors overall and by type of myopia is shown in Table 1. There were important and anticipated variations overall with respect to sex (higher proportion of women had childhood-onset myopia, S2 Fig), highest educational attainment (the overall proportion with no educational qualifications declined over time from 30% to 5%, S3 Fig), socio-economic status (accommodation tenure) and ethnicity. Notably 47.4% of those of Chinese ethnicity had myopia (almost twice the frequency of any other ethnic group), mainly childhood-onset.

### Associations of myopia with socio-demographic and environmental risk factors

Fully adjusted analysis, to model associations between myopia (all and by onset) and socio-demographic factors, showed later year of birth (younger cohort) was associated with reduced odds of myopia overall, but this was driven by a progressive decline (independent protective effect) in odds of childhood-onset myopia across all cohorts, resulting in an approximately 30% reduction in risk of childhood-onset myopia to those born from the 1960s onwards compared to the baseline cohort 1 (born between 1939 and 1944). By comparison, the odds of adult-onset myopia increased in cohorts 2 to 5 (Table 2). Females were at increased independent risk of myopia overall, but this comprised 24% increased risk of childhood-onset and a 12% decreased risk of adult-onset myopia (Table 2). Chinese ethnicity was associated with a 90% increased risk of myopia overall but specifically a 19% increased risk of adult-onset and 240% increased risk of childhood-onset myopia compared to White ethnicity, with all other ethnic groups being ‘protective’. Highest educational attainment was strongly associated with increased risk of childhood-onset myopia, with a clear ‘dose response’ relationship. There was a similar gradient, with smaller effect sizes, for adult-onset myopia. Thus, the largest effect sizes for associations with educational attainment after the age of 18 years in both types, with a greater effect size for males (S2 and S3 Tables). This is compatible with capturing the highest educational intensity/experience during school as either a ‘trajectory’ or ‘threshold’ effect with respect to myopia risk. Higher current socio-economic status (using the conventional index of housing tenure) was associated with increased risk of adult-onset myopia with a gradient across categories whereas only highest category of socio-economic status was associated with childhood-onset myopia.

**Table 2:** Association of myopia (all, childhood-onset and adult-onset), with socio-demographic and environmental factors in 75,297 participants.

Factors	Childhood-onset myopia			Adult-onset myopia			All myopia			Emmetropia
	n	Crude OR*	Adjusted OR **	n	Crude OR*	Adjusted OR **	n	Crude OR*	Adjusted OR **	n
	16,389		(95% CI)	11,685		(95% CI)	28,074		(95% CI)	47,223
Year of birth										
1939–1944	2,468	1	1	1,436	1	1	3,904	1	1	6,639
1945–1949	4,132	**1.11**	1.03 (0.98, 1.08)	2,392	**1.10**	**1.07 (1.03, 1.12)**	6,524	**1.11**	**1.04 (1.00, 1.09)**	10,034
1950–1954	3,313	**1.09**	0.98 (0.87, 1.10)	2,143	**1.21**	**1.16 (1.08, 1.26)**	5,456	**1.14**	1.04 (0.96, 1.13)	8,164
1955–1959	2,672	**0.92**	**0.85 (0.76, 0.96)**	2,213	**1.31**	**1.26 (1.22, 1.30)**	4,885	**1.06**	1.00 (0.94, 1.07)	7,823
1960–1964	2,114	**0.73**	**0.70 (0.62, 0.79)**	2,022	**1.21**	**1.17 (1.09, 1.25)**	4,136	**0.91**	**0.87 (0.80, 0.94)**	7,753
1965–1970	1,690	**0.67**	**0.64 (0.55, 0.75)**	1,479	1.00	0.97 (0.93, 1.01)	3,169	**0.79**	**0.76 (0.70, 0.82)**	6,810
Sex										
Male	6,929	1	1	5,821	1	1	12,750	1	1	22,246
Female	9,460	**1.22**	**1.24 (1.13, 1.35)**	5,864	**0.90**	**0.88 (0.84, 0.93)**	15,324	**1.07**	**1.08 (1.01, 1.15)**	24,977
Highest educational qualification ¥										
None	984	1	1	1,025	1	1	2,009	1	1	6,738
O-level	3,426	**1.73**	**1.91 (1.75, 2.09)**	3,269	**1.58**	**1.52 (1.38, 1.66)**	6,695	**1.65**	**1.72 (1.58, 1.88)**	13,590
A-level	2,793	**2.18**	**2.39 (2.17, 2.64)**	2,201	**1.65**	**1.57 (1.43, 1.72)**	4,994	**1.91**	**1.97 (1.85, 2.10)**	8,783
Higher-level	9,186	**3.47**	**3.82 (3.63, 4.02)**	5,190	**1.88**	**1.80 (1.66, 1.95)**	14,376	**2.66**	**2.76 (2.62, 2.90)**	18,112
Accommodation tenure										
Council rental	759	1	1	615	1	1	1,374	1	1	3,584
Private rental	564	**1.18**	0.99 (0.89, 1.10)	442	**1.14**	1.04 (0.92, 1.19)	1,006	**1.17**	1.01 (0.90, 1.13)	2,252
Own with mortgage	5,888	**1.45**	1.09 (0.90, 1.33)	5,002	**1.52**	**1.33 (1.13, 1.56)**	10,890	**1.48**	**1.19 (1.00, 1.41)**	19,207
Own	9,178	**1.95**	**1.28 (1.04, 1.57)**	5,626	**1.48**	**1.36 (1.14, 1.63)**	14,804	**1.74**	**1.31 (1.09, 1.58)**	22,180
Ethnicity										
White	15,024	1	1	10,455	1	1	25,479	1	1	41,731
Mixed ethnicity	163	0.94	1.04 (0.83, 1.31)	119	0.99	1.02 (0.89, 1.17)	282	0.96	1.03 (0.89, 1.19)	480
Asian or Asian British§	499	**0.70**	**0.78 (0.71, 0.86)**	433	**0.87**	**0.87 (0.80, 0.98)**	932	**0.77**	**0.82 (0.75, 0.90)**	1,983
Black or Black British	380	**0.53**	**0.62 (0.53, 0.73)**	447	**0.89**	**0.95 (0.92, 0.99)**	827	**0.67**	**0.76 (0.70, 0.84)**	2,008
Chinese	166	**2.40**	**2.33 (1.72, 3.16)**	57	**1.18**	**1.20 (1.04, 1.38)**	223	**1.90**	**1.83 (1.43, 2.36)**	192
Other	157	**0.53**	**0.56 (0.47, 0.66)**	174	**0.84**	**0.90 (0.82, 0.99)**	331	**0.65**	**0.70 (0.62, 0.79)**	829

*OR = Odds Ratio. 95% CI = 95% confidence interval.

** Adjusted by year of birth, sex, educational qualification, accommodation tenure, ethnicity, variance adjustment for test centre. ¥O level: State examination at age 16 years; A level: State examination at age 18 years. §Asian category includes Indian, Pakistani and Bangladeshi. **Bold** fonts indicate significant associations at the 5% level. For this analysis we have excluded 2,515 with missing data in the selected factors (as detailed in Table 1).

### Effect modification (interaction) by sociodemographic and environmental risk factors

**Fig 4:**
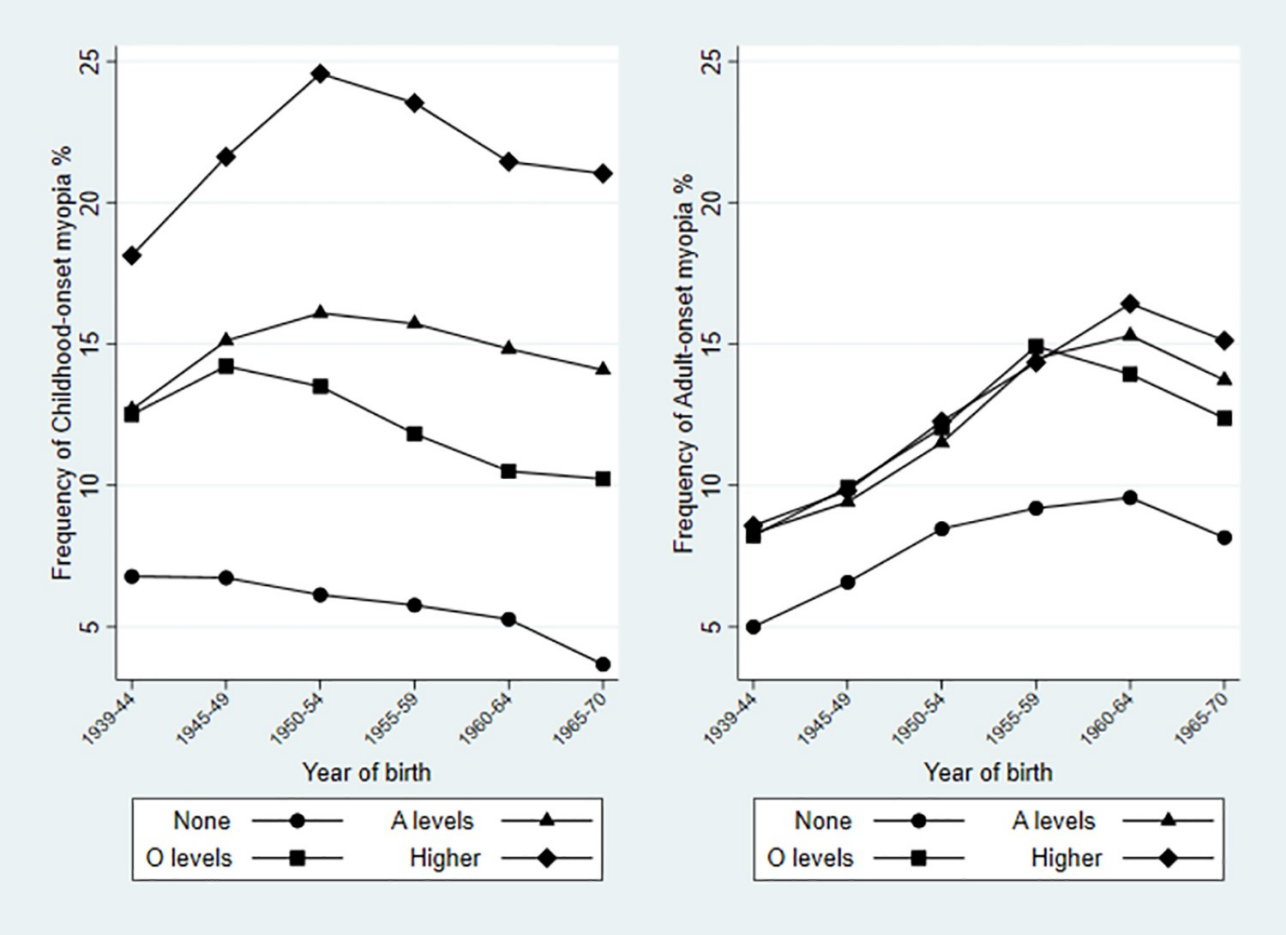
Frequency of (a) childhood-onset and (b) adult-onset myopia by highest educational attainment*, by year of birth as 5-year bands. *No qualifications, ‘O’ levels (examinations at statutory school-leaving age in the UK), ‘A’ levels (examinations at age 18 years in the UK) or higher level of education.

Effect modification was examined by adding interaction terms to the fully adjusted model reported in Table 2. Evidence of effect modification with highest educational attainment between sex (p<0.001), year of birth (p<0.001) and ethnicity (p<0.001) was observed. Table 3 and Fig 4 show a clear temporal trend of increasing magnitude of adjusted association of higher educational attainment (evident for each category of educational attainment) for childhood-onset myopia, notably in the association with the top category of post-school higher education, OR 3.3 [2.8, 4.0] in cohort 6 (youngest) compared to OR 8.0 [4.2, 13] in cohort 1 (oldest). By contrast the size of the associations for adult-onset myopia remained largely unchanged (with overlapping 95% confidence intervals) over time. Interestingly, an increasingly stronger protective effect of having no educational qualifications (plausibly capturing reduced educational experience or intensity during childhood) over time, was attributable to the relationship with childhood-onset myopia, with this protective effect increasing over time—from 15% risk reduction in cohort 1 compared to 77% risk reduction in the cohort 6. Taken together these changes over time in the associations with education are compatible with a cohort effect in gene-environment interaction in myopia causation.

**Table 3:** Interactions in associations with myopia (all, childhood-onset and adult-onset) between year of birth and no educational qualifications.

		Educational attainment adjusted Odds Ratios (95% confidence interval)			
		No qualification	O level	A level	Higher level
**All myopia, n = 28,074**					
Year of birth					
1939–44		1	**1.91 (1.73, 2.10)**	**1.91 (1.65, 2.20)**	**2.70 (2.51, 2.90)**
1945–49	vs 1939–44	0.97 (0.90, 1.05)	0.99 (0.86, 1.15)	1.08 (0.99, 1.18)	1.10 (0.98, 1.23)
	vs No qualification	1	**1.95 (1.72, 2.21)**	**2.12 (1.86, 2.41)**	**3.06 (2.72, 3.43)**
1950–54	vs 1939–44	0.92 (0.75, 1.13)	0.94 (0.68, 1.30)	1.02 (0.77, 1.35)	1.04 (0.83, 1.31)
	vs No qualification	1	**1.87 (1.45, 2.41)**	**2.20 (1.73, 2.80)**	**3.47 (2.77, 4.35)**
1955–59	vs 1939–44	0.86 (0.69, 1.07)	0.88 (0.65, 1.18)	0.96 (0.76, 1.20)	0.98 (0.84, 1.14)
	vs No qualification	1	**1.94 (1.61, 2.35)**	**2.33 (1.95, 2.80)**	**3.50 (2.81, 4.36)**
1960–64	vs 1939–44	0.77 (0.58, 1.03)	0.79 (0.62, 1.19)	0.86 (0.62, 1.19)	0.88 (0.69, 1.11)
	vs No qualification	1	**1.71 (1.34, 2.17)**	**2.37 (1.77, 3.17)**	**3.52 (2.59, 4.78)**
1965–70	vs 1939–44	**0.58 (0.49, 0.68)**	**0.59 (0.44, 0.80)**	**0.64 (0.48, 0.86)**	**0.65 (0.51, 0.84)**
	vs No qualification	1	**1.96 (1.59, 2.41)**	**2.69 (2.23, 3.25)**	**4.17 (3.32, 5.22)**
**Adult-onset myopia, n = 11,685**					
Year of birth					
1939–44		1	**1.81 (1.44, 2.29)**	**1.77 (1.44, 2.19)**	**2.05 (1.81, 2.32)**
1945–49	vs 1939–44	1.14 (0.98, 1.32)	1.03 (0.87, 1.22)	1.06 (0.96, 1.17)	1.08 (0.96, 1.20)
	vs No qualification	1	**1.64 (1.47, 1.83)**	**1.65 (1.51, 1.80)**	**1.94 (1.76, 2.14)**
1950–54	vs 1939–44	1.24 (0.96, 1.62)	1.12 (0.82, 1.55)	1.16 (0.83, 1.62)	1.18 (0.88, 1.57)
	vs No qualification	1	**1.54 (1.17, 2.03)**	**1.61 (1.22, 2.12)**	**2.03 (1.50, 2.75)**
1955–59	vs 1939–44	**1.22 (1.02, 1.45)**	1.10 (0.74, 1.64)	1.13 (0.90, 1.43)	1.15 (0.96, 1.38)
	vs No qualification	1	**1.77 (1.36, 2.31)**	**1.86 (1.44, 2.40)**	**2.19 (1.74, 2.76)**
1960–64	vs 1939–44	1.13 (0.83, 1.53)	1.02 (0.69, 1.50)	1.05 (0.79, 1.39)	1.07 (0.87, 1.31)
	vs No qualification	1	**1.52 (1.15, 2.00)**	**1.92 (1.39, 2.63)**	**2.43 (1.71, 3.44)**
1965–70	vs 1939–44	0.88 (0.67, 1.14)	0.80 (0.58, 1.10)	0.82 (0.58, 1.15)	0.83 (0.61, 1.13)
	vs No qualification	1	**1.59 (1.24, 2.04)**	**2.02 (1.55, 2.65)**	**2.63 (2.03, 3.41)**
**Childhood-onset myopia, n = 16,389**					
Year of birth					
1939–44		1	**1.99 (1.77, 2.36)**	**2.06 (1.68, 2.52)**	**3.33 (2.77, 3.99)**
1945–49	vs 1939–44	0.85 (0.72, 1.00)	0.97 90.81, 1.16)	1.09 (0.99, 1.20)	1.12 (0.99, 1.25)
	vs No qualification	1	**2.27 (1.90, 2.72)**	**2.66 (2.25, 3.11)**	**4.37 (3.92, 4.87)**
1950–54	vs 1939–44	**0.67 (0.53, 0.85)**	0.76 (0.50, 1.17)	0.86 (0.61, 1.20)	0.88 (0.67, 1.15)
	vs No qualification	1	**2.37 (1.91, 2.94)**	**3.13 (2.49, 3.93)**	**5.80 (4.59, 7.34)**
1955–59	vs 1939–44	**0.58 (0.43, 0.78)**	0.66 (0.42, 1.06)	0.74 (0.49, 1.14)	0.76 (0.53, 1.09)
	vs No qualification	1	**2.26 (1.71, 2.99)**	**3.21 (2.66, 3.88)**	**5.96 (4.27, 8.30)**
1960–64	vs 1939–44	**0.49 (0.36, 0.68)**	**0.56 (0.33, 0.95)**	**0.63 (0.41, 0.97)**	**0.65 (0.45, 0.92)**
	vs No qualification	1	**2.07 (1.55, 2.77)**	**3.32 (2.40, 4.57)**	**5.83 (4.05, 8.40)**
1965–70	vs 1939–44	**0.33 (0.26, 0.41)**	**0.37 (0.26, 0.53)**	**0.42 (0.31, 0.57)**	**0.43 (0.34, 0.55)**
	vs No qualification	1	**2.85 (1.74, 4.69)**	**4.36 (2.65, 7.22)**	**8.04 (4.19, 13.1)**

*Adjusted by sex vs educational qualification interaction, accommodation tenure, ethnicity, variance adjustment by test centre. **Bold** fonts indicate significant within cohort associations at the 5% level.

The association between higher educational attainment and myopia risk was evident for all ethnic groups in relation to childhood-onset myopia, and in a gradient with the category of higher qualifications after the age of 18 years having the strongest effect size except amongst Black/Black British participants (Table 4). By contrast the association between higher educational attainment and adult-onset myopia was only evident for White and Asian ethnicities, despite similar sample sizes between the latter and participants of African ancestry. No educational qualifications appeared to have a greater protective effect amongst those from ethnic minority populations versus White participants, although the smaller sample sizes resulted in wide confidence intervals for the effect estimates. Finally, despite the limited sample size, there was a striking interaction between Chinese ethnicity and education which could reflect the relationship between genetic predisposition and environment-environment interactions: Chinese participants without any educational qualifications were not at increased risk of either childhood-onset or adult-onset myopia whilst those with higher highest educational qualifications had the highest risk of both. Together, these findings provide indirect evidence of gene-environment interaction, in particular for childhood-onset myopia.

**Table 4:** Interactions in associations with myopia (all, childhood-onset and adult -onset) between ethnicity and no educational qualifications.

		Educational attainment adjusted Odds Ratios (95% confidence interval)			
		No qualification	O level	A level	Higher level
**All myopia, n = 28,074**					
Ethnicity					
White		1	**1.67 (1.53, 1.82)**	**1.96 (1.82, 2.11)**	**2.74 (2.60, 2.89)**
Mixed	vs White	1.16 (0.81, 1.67)	1.06 (0.81, 1.39)	0.92 (0.58, 1.46)	1.05 (0.91, 1.21)
	vs No qualification	1	1.52 (0.97, 2.40)	1.54 (0.75, 3.18)	**2.47 (1.56, 3.91)**
Asian/British Asian	vs White	**0.61 (0.47, 0.81)**	**0.85 (0.76, 0.96)**	0.89 (0.75, 1.06)	**0.82 (0.72, 0.94)**
	vs No qualification	1	**2.34 (1.59, 3.43)**	**2.85 (2.30, 3.54)**	**3.70 (2.61, 5.25)**
Black/Black British	vs White	0.76 (0.47, 1.24)	**1.24 (1.11, 1.38)**	**0.67 (0.54, 0.83)**	**0.60 (0.56, 0.66)**
	vs No qualification	1	**2.72 (1.73, 4.27)**	1.72 (0.95, 3.13)	**2.18 (1.43, 3.33)**
Chinese	vs White	1.13 (0.57, 2.23)	1.66 (0.90, 3.07)	**1.66 (1.46, 1.89)**	**2.06 (1.67, 2.55)**
	vs No qualification	1	**2.46 (1.27, 4.74)**	**2.89 (1.42, 5.88)**	**5.00 (2.76, 9.05)**
Other	vs White	**0.61 (0.46, 0.81)**	**0.72 (0.53, 0.99)**	**0.59 (0.47, 0.74)**	**0.72 (0.65, 0.81)**
	vs No qualification	1	**1.98 (1.28, 3.09)**	**1.89 (1.21, 2.95)**	**3.26 (2.29, 4.63)**
**Adult-onset myopia, n = 11,685**					
Ethnicity**					
White		1	**1.49 (1.35, 1.64)**	**1.58 (1.44, 1.74)**	**1.77 (1.63, 1.92)**
Mixed	vs White	1.32 (0.52, 3.34)	1.04 (0.85, 1.28)	**0.68 (0.50, 0.93)**	1.12 (0.90, 1.39)
	vs No qualification	1	1.17 (0.50, 2.75)	0.82 (0.25, 2.65)	1.49 (0.51, 4.36)
Asian/British Asian	vs White	0.63 (0.37, 1.07)	**0.89 (0.82, 0.97)**	**0.82 (0.69, 0.97)**	0.96 (0.79, 1.16)
	vs No qualification	1	**2.11 (1.13, 3.91)**	**2.06 (1.26, 3.35)**	**2.69 (1.46, 4.96)**
Black/Black British	vs White	0.90 (0.60, 1.37)	**1.21 (1.04, 1.41)**	**0.70 (0.66, 0.74)**	**0.92, (0.87, 0.98)**
	vs No qualification	1	**2.00 (1.26, 3.16)**	1.23 (0.80, 1.90)	**1.80 (1.24, 2.63)**
Chinese	vs White	1.13 (0.40, 3.21)	0.97 (0.47, 2.00)	**0.38 (0.23, 0.61)**	**1.55 (1.41, 1.71)**
	vs No qualification	1	1.27 (0.35, 4.59)	0.53 (0.12, 2.28)	2.42 (0.78, 7.51)
**Childhood-onset myopia, n = 16,389**					
Ethnicity**					
White		1	**1.82 (1.69, 1.97)**	**2.33 (2.11, 2.58)**	**3.78 (3.61, 3.96)**
Mixed	vs White	0.93 (0.44, 2.92)	1.05 (0.70, 1.58)	1.11 (0.63, 1.95)	1.01 (0.86, 1.19)
	vs No qualification	1	2.06 (0.75, 5.66)	**2.78 (1.70, 4.53)**	**4.11 (2.11, 8.01)**
Asian/British Asian	vs White	**0.56 (1.07, 1.96)**	0.81 (0.65, 1.01)	0.95 (0.75, 1.20)	**0.75 (0.68, 0.84)**
	vs No qualification	1	**2.64 (1.92, 3.64)**	**3.93 (3.02, 5.12)**	**5.09 (4.36, 5.94)**
Black/Black British	vs White	**0.57 (1.16, 4.16)**	**1.25 (1.09, 1.42)**	**0.63 (0.44, 0.91)**	**0.42 (0.36, 0.49)**
	vs No qualification	1	**4.00 (2.26, 7.10)**	**2.59 (1.11, 6.05)**	**2.78 (1.58, 4.90)**
Chinese	vs White	**1.07 (1.28, 3.54)**	**2.28 (1.24, 4.22)**	**2.79 (2.24, 3.43)**	**2.38 (1.78, 3.18)**
	vs No qualification	1	**3.88 (2.36, 6.38)**	**6.05 (2.38, 15)**	**8.37 (4.09, 17)**

*Adjustment by year of birth, sex, accommodation tenure, variance adjustment for test centre.

**Estimates by Other ethnicity cannot be made due to low numbers. **Bold** fonts indicate significant within cohort associations at the 5% level.

## Discussion

A moderate increase in myopia frequency over three decades in the UK took the overall baseline from 20.0% of older adults in cohort 1 to 29.2% of the youngest, in cohort 6. However temporal trends varied by type of myopia; with a substantially greater increase in adult-onset (doubling) and in low myopia, as well as plateauing of frequency a decade later (those born in 1960s) for adult-onset myopia. By comparison, a clinically important shift in distribution resulting in increase in severity was only observed in childhood-onset myopia. There were notable differences in patterns of associations with sex, ethnicity, socio-economic status and education by type of myopia along with complex interactions (effect modification) between these predictors over time. Notably the strong positive association across increasing levels of higher educational attainment (proxy for prior educational experience/intensity) and the negative (protective) effect of no educational qualifications was greater for childhood-onset myopia. Amplification of these associations over time (effect modification), more so in men, was substantially greater for childhood-onset than adult-onset myopia indicating greater cumulative experience in those with childhood-onset myopia. Importantly the observed associations with education did not account fully for the cohort effect in myopia frequency and observed interactions between ethnicity and education.

The design and scale of UK Biobank afforded the opportunity to carefully dissect temporal trends taking account of type of myopia, enabling a more meaningful analysis of associations with key risk factors than is achieved by combining all myopia. Standardised ophthalmic assessment and structured standardised collection of information on key environmental factors enabled high quality data to be collected. Using our systematic approach [21] to combining refraction data and age at first optical correction together with self-report on eye conditions enabled both exclusion of those with myopia as a secondary condition and categorisation of type of primary myopia in the remaining individuals. The potential for misclassification using non-cycloplegic autorefraction [22] is low in the age group in our study and its effects minimal given our use of the recently accepted consensus threshold for clinically meaningful myopia of -1.0D [19, 20, 23, 24]. Whilst significant efforts have been directed to measuring educational intensity and experience for example using diaries or ‘wearable’ devices, proxy measures of educational intensity/exposure such as years of education are conventionally used in large scale whole population studies when myopia research has not been the sole/primary purpose. We used educational attainment (lifetime highest educational qualifications), as a proxy for educational experience and intensity, as it directly captures a combination of years of schooling along with childhood time spent on educational activities outside of school (e.g. homework) and educational activities into adult life. We analysed childhood-onset and adult-onset myopia separately, partly to address the potential for confounding by parental history of myopia, which was not measured in Biobank. As UK Biobank comprises volunteers, population prevalence estimates cannot be estimated but the overall frequency of primary myopia in the UKBB is consistent with other European studies, taking into account differences in age range and threshold values for refractive errors between studies [25, 26]. Moreover, risk factor associations in UK Biobank are accepted to be generalisable [27] but as in any cross-sectional study, the possibility of residual confounding exists, so associations reported here do not imply causality.

Conventionally temporal trends and cohort effects are investigated using longitudinal data, but we exploited the scope of the UK Biobank to create historical cohorts of adults who had also fortuitously been assessed at an age when the final myopia phenotype, comprising both type and severity of myopia, could be reliably assigned. As such there are no studies with which we can directly compare our findings. The findings of the present study are consistent with the findings of our prior research in the population-based UK 1958 British birth cohort at age 44 years with respect to frequency (after accounting for small differences in myopia definition) [14, 19], the strong predominance of adult-onset myopia and strikingly similar associations with sex, social class and educational attainment and additionally cognitive performance during childhood. Furthermore, temporal trends observed in the present study are comparable to those reported in a meta-analysis of heterogeneous European studies in terms of both point prevalence and increase in prevalence of all myopia comparing those born by 1939 versus by 1970 [26] This supports the generalisability of our findings.

Myopia is a multifactorial trait and numerous environmental factors e.g. prenatal growth and maternal health during pregnancy, cumulative education/near work activities, socioeconomic status and time spent outdoors are all reported to involved [5, 13, 14, 28–33]. We report here evidence that supports a ‘complex’ of changing environmental factors and presumably changing gene-environment interactions, accounting for increasing overall myopia frequency and shift in distribution to increase in the UK. We show that differential patterns by type of myopia underlying these temporal trends. For example, by finding of interactions between cohort (year of birth) and sex which may explain the conflicting prior reports on all myopia and sex [6, 26, 34–36]. Equally our study shows that the shift in median MSE (increasing severity) over time occurred only for childhood onset (and in particular onset before 10 years), by contrast with adult-onset myopia which increased in frequency but the average MSE remained stable over time. Our findings regarding the different eras for peak/plateau of risk of childhood-onset versus adult-onset myopia could be analogous to different ‘waves’ in a long-duration epidemic. For example, the peak of childhood-onset representing the impact of universal schooling and raising of the school leaving and broader changes in maternal and child health, whilst the ongoing increase in risk of adult-onset myopia reflecting the broader changes in lifestyles with more time spent indoors, increased near viewing occupations or activities.

Interesting variations by ethnicity were evident in the relationship between year of birth and education but only for childhood-onset myopia in UK Biobank. Interpretation of findings relating to UK Biobank participants of Chinese ethnicity– 80.5% of whom were born outside the UK and immigrated aged 17 years or older–is difficult because information is lacking about relevant childhood exposures and also, because they frequently (58.5%) had a degree/higher qualification. The present myopia boom in Asian populations originated after the period studied in Biobank but it is noteworthy that most of the Chinese participants in our study had attained higher education qualifications prior to this epidemic. Ongoing investigations of other ethnic groups born in the UK have not yet demonstrated important gene-environment interaction in myopia frequency in the UK [37].

Since Kepler in the early 17th century, aetiological research has been especially directed to the role of retinal blur (defocus) affecting the peripheral retina during “near work” viewing, such as in an educational context [38, 39], with a more recent focus on the active protective effect of ‘distance’ viewing achieved during time spent outdoors [40] and data-driven approaches to distinguishing the two [41]. Since it would be unethical to undertake randomised clinical trials of different levels of education provision, recent studies have utilised Mendelian randomisation and findings support a causal relationship between a more time spent in education and subsequent myopia [42, 43]. Nevertheless, the association with education is not the sole cause of increasing myopia prevalence [6, 8]. For example, myopia in older populations in China is primarily early-onset ‘genetic’ myopia and independent of educational experience, whereas environmentally-induced myopia associated with education parameters is seen in more recent cohorts [44]. This aligns with our finding that adult-onset myopia accounted for the majority of increase in myopia frequency in the present study, was positively associated with more time in education (although nevertheless to a lesser degree than for childhood-onset) but the effect size of the association changed little over time. By contrast, the ‘protective’ effect of minimal educational experience (no lifetime qualifications) was only evident for childhood-onset myopia and increasingly so over time. Together these findings point to other environmental factors, related to complex societal and behavioural changes that occur with urbanisation, having a differential influence the development of adult-onset myopia which predominates in the UK today.

The childhoods captured in UK Biobank occurred during important changes in education, health and nutrition. Older study participants were children during World War II when many aspects of life including education and access to a varied diet were disrupted/limited. Prior to the formation of the NHS in 1948 and the introduction of vision screening and free prescription glasses affected children may not have been tested or worn glasses [45]. There has been a shift over time in the proportion of children opting to stay in higher and further education and, in parallel, changing methods of teaching, widespread use of TV and more recently the widespread use of electronic screen devices and extension of such activities into free time has altered the pattern of near work activity. In future, although the prevalence of myopia may increase the association between myopia and education parameters may not be as strong and educational attainment as a marker of socioeconomic status and lifestyle may not be a singularly useful marker. The changing lifestyles of children, from pre-school to school age, is raising concerns about health and well-being including about the potential impact of use of screen devices on sleep patterns and cognitive development [46]. In the UK, as part of a public health strategies for children [47–49] physical activity initiatives have been introduced which as a by-product of balancing indoor (near viewing) and outdoor (distance viewing) activity, may help curb the risk of myopia.

Our demonstration of complex temporal trends in the UK, points to a dynamic complex of environmental risk factors and cohort effects in gene-environment interactions, discernible only by distinguishing by between childhood and adult-onset myopia. The evidence of historical plateauing of frequency suggests that stabilisation or reversal of temporal trends is possible. Evidence of a differential impact of educational experience/intensity on childhood-onset and high myopia and its amplification over time predicts future increasing frequency of childhood-onset myopia without attention to educational intensity/experience continues to increase. But attention to childhood-onset myopia alone will not address the considerable public health impact of myopia. Whilst adult-onset myopia is generally less severe, it remains as/more common in the UK population and thus confers considerable personal and societal burden. A mixed economy of research is needed to improve our understanding of modifiable risk factors across the life course and how to tackle them.

## Supporting information

S1 Fig Flowchart of participation. (PDF)

S2 Fig Frequency of childhood-onset and adult-onset myopia by sex and by year of birth as 5-year bands. (PDF)

S3 Fig Distribution of highest educational attainment by year of birth as 5 year bands. (PDF)

S4 Fig Frequency of childhood-onset and adult-onset myopia by ethnicity, by year of birth as 5-year bands. (PDF)

S1 Table Median MSE (mean spherical equivalent) and interquartile range (IQR): Childhood-onset myopia (subdivided as before age 10 and between aged 10 and 16 years) and all childhood-onset myopia, by year of birth as 5-year bands. * Numbers do not include n = 120 individuals who did not report age of first wearing glasses and were assigned category based on other aggregate evidence.
(PDF)

S2 Table Association of myopia (all, childhood-onset and adult-onset), with socio-demographic and environmental factors in females. * OR = Odds Ratio. 95% CI = 95% confidence interval. ** Adjusted by year of birth, sex, educational qualification, accommodation tenure, ethnicity, variance adjustment for test centre. ¥O level: State examination at age 16 years; A level: State examination at age 18 years. §Asian category includes Indian, Pakistani and Bangladeshi. **Bold** fonts indicate significant associations at the 5% level.
(PDF)

S3 Table Association of myopia (all, childhood-onset and adult-onset), with socio-demographic and environmental factors in males. *OR = Odds Ratio. 95% CI = 95% confidence interval. ** Adjusted by year of birth, sex, educational qualification, accommodation tenure, ethnicity, variance adjustment for test centre. ¥O level: State examination at age 16 years; A level: State examination at age 18 years. §Asian category includes Indian, Pakistani and Bangladeshi. **Bold** fonts indicate significant associations at the 5% level.
(PDF)

S4 Table Frequency of myopia (all, childhood-onset and adult-onset), emmetropia and hypermetropia in the UK Biobank population: Distribution of socio-demographic and environmental factors. ¥O level: State examination at age 16 years; A level: State examination at age 18 years. §Asian category includes Indian, Pakistani and Bangladeshi.
(PDF)

S5 Table Frequency of myopia (all, childhood-onset and adult-onset), emmetropia and hypermetropia: Distribution of socio-demographic and environmental factors, by year of birth (1939–44, 1945–49, 1950–54, 1955–59, 1960–64 and 1965–69). ¥O level: State examination at age 16 years; A level: State examination at age 18 years. §Asian category includes Indian, Pakistani and Bangladeshi.
(PDF)
