# Supplementary figures and images for: Temporal trends in frequency, type and severity of myopia and associations with key environmental risk factors in the UK: Findings from the UK Biobank Study
Source: PLoS One. 2022 Jan 19;17(1):e0260993. doi: 10.1371/journal.pone.0260993 (PMC8769366; doi:10.1371/journal.pone.0260993)

**S1 fig: Flowchart of participation**

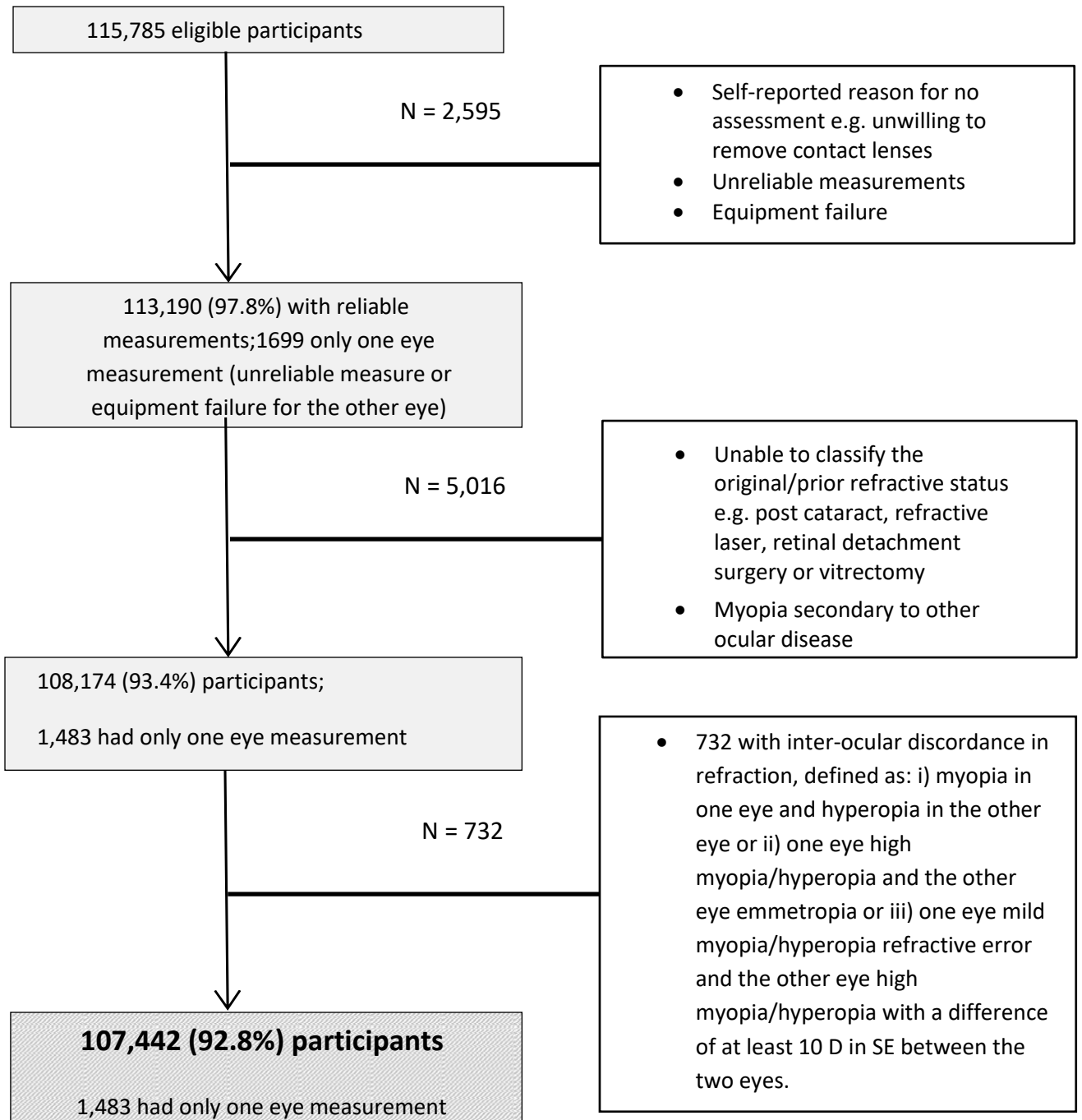

Supplement: S1 Fig — (PDF) [file pone.0260993.s001.pdf]

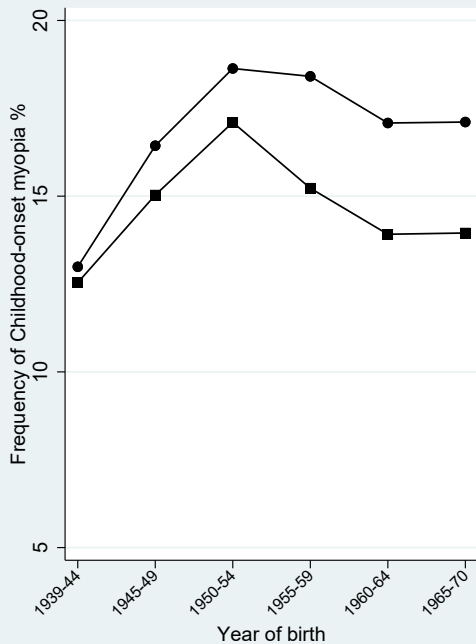

Female —●— Male —■—

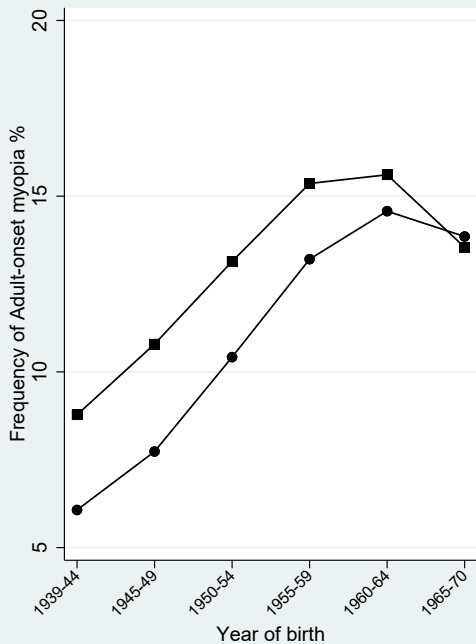

Female —●— Male —■—

Supplement: S2 Fig — (PDF) [file pone.0260993.s002.pdf]

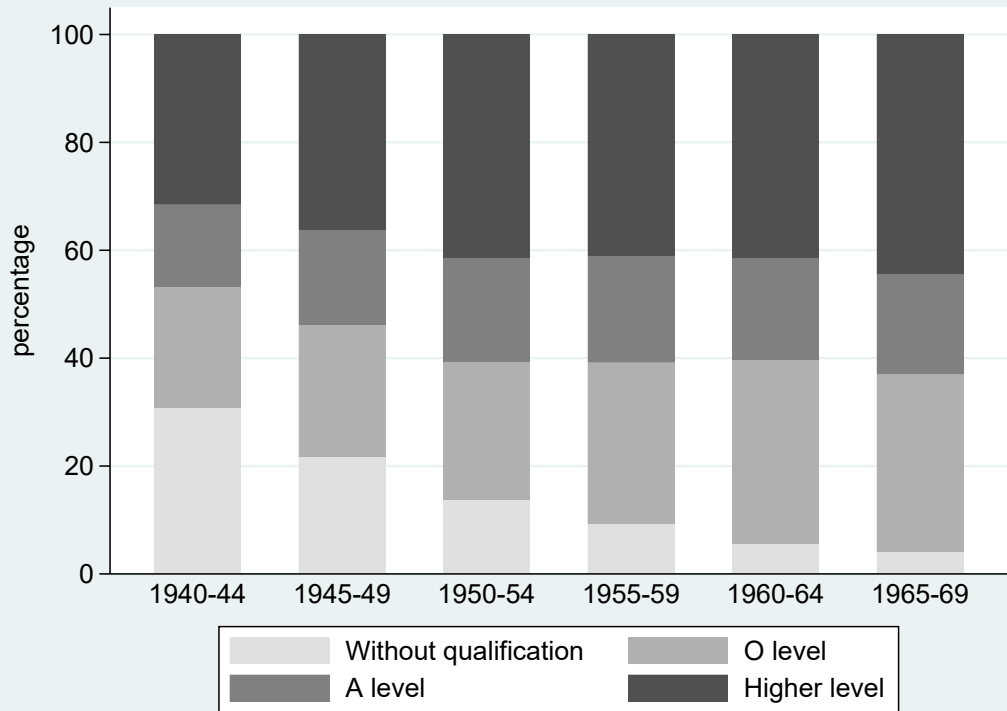

Supplement: S3 Fig — (PDF) [file pone.0260993.s003.pdf]

# Childhood-onset myopia

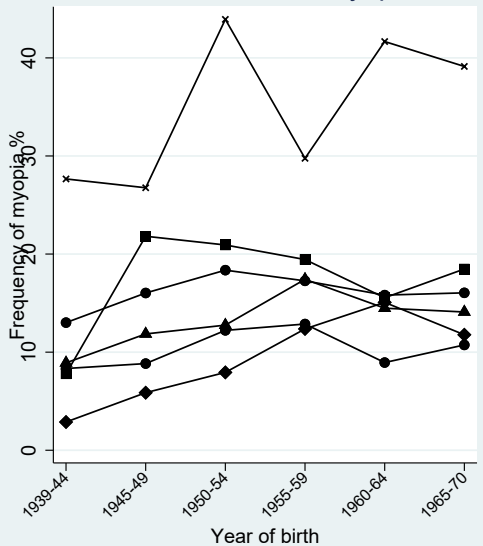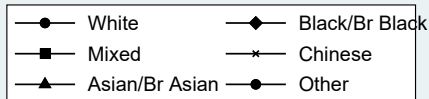

# Adult-onset myopia

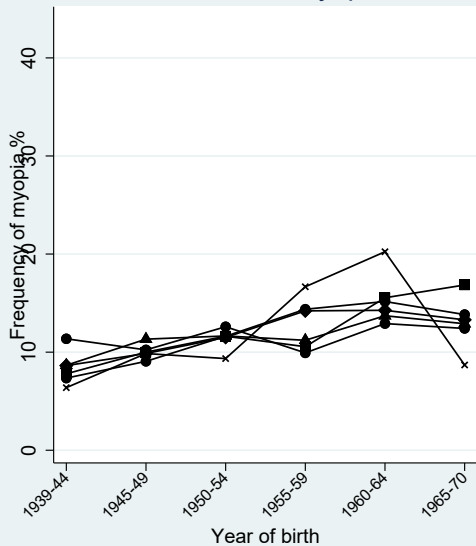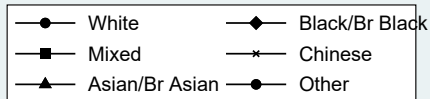

Supplement: S4 Fig — (PDF) [file pone.0260993.s004.pdf]
