# Supplementary material for: Temporal trends in frequency, type and severity of myopia and associations with key environmental risk factors in the UK: Findings from the UK Biobank Study
Source: PLoS One. 2022 Jan 19;17(1):e0260993. doi: 10.1371/journal.pone.0260993 (PMC8769366; doi:10.1371/journal.pone.0260993)
Supplement: S1 Table — * Numbers do not include n = 120 individuals who did not report age of first wearing glasses and were assigned category based on other aggregate evidence. (PDF) [file pone.0260993.s005.pdf]

S1_Table: Median MSE (mean spherical equivalent) and interquartile range (IQR): childhood-onset myopia (subdivided as before age 10 and between aged 10 and 16 years) and all childhood-onset myopia, by year of birth as 5 year bands.

	Childhood-onset <10yrs		Childhood-onset 10 to <16yrs		All childhood-onset myopia	
Year of birth	n	Median MSE IQR	n	Median MSE IQR	n	Median MSE IQR
1939-44	594	-4.66 [-2.97, -6.65]	1,681	-3.57 [-2.25, -4.96]	2,275	-3.80 [-2.39, -5.44]
1945-49	1,117	-4.94 [-3.15, -7.17]	2,745	-3.57 [-2.31, -5.18]	3,862	-3.88 [-2.52, -5.70]
1950-54	935	-5.36 [-3.57, -7.58]	2,143	-3.84 [-2.45, -5.44]	3,078	-4.18 [-2.72, -6.04]
1955-59	777	-5.64 [-3.63, -7.50]	1,658	-4.03 [-2.69, -5.58]	2,435	-4.39 [-2.97, -6.34]
1960-64	580	-5.92 [-3.79,-8.14]	1,336	-3.98 [-2.66, -5.55]	1,916	-4.46 [-2.98, -6.37]
1965-70	444	-5.43 [-3.79, -7.78]	1,126	-4.07 [-2.77, -5.65]	1,570	-4.43 [-2.98, -6.18]
Total	4,447	-5.27 [-3.43, -7.45]	10,687	-3.80 [-2.46, -5.36]	15,134	-4.14 [-2.67, -5.99]

- Numbers do not include n=120 individuals who did not report age of first wearing glasses and were assigned category based on other aggregate evidence.
