# Supplementary material for: Temporal trends in frequency, type and severity of myopia and associations with key environmental risk factors in the UK: Findings from the UK Biobank Study
Source: PLoS One. 2022 Jan 19;17(1):e0260993. doi: 10.1371/journal.pone.0260993 (PMC8769366; doi:10.1371/journal.pone.0260993)
Supplement: S2 Table — * OR = Odds Ratio. 95% CI = 95% confidence interval. ** Adjusted by year of birth, sex, educational qualification, accommodation tenure, ethnicity, variance adjustment for test centre. ¥O level: State examination at age 16 years; A level: State examination at age 18 years. §Asian category includes Indian, Pakistani and Bangladeshi. Bold fonts indicate significant associations at the 5% level. (PDF) [file pone.0260993.s006.pdf]

S2.1_Table: Association of myopia (all, childhood-onset and adult-onset), with socio-demographic and environmental factors in **females.**

	Childhood-onset myopia			Adult-onset myopia			All myopia			Emmetropia
Factors	N 9460	Unadjusted OR* (95% CI)	Adjusted OR ** (95% CI)	N 5864	Unadjusted OR* (95%CI)	Adjusted OR ** (95% CI)	N 15324	Unadjusted OR* (95%CI)	Adjusted OR ** (95% CI)	N 24977
Year of birth	N			n			n			N
1939-1944	1254	**1**	1	586	**1**	**1**	1840	**1**	1	3083
1945-1949	2321	1.10	**1.03 (1.02, 1.05)**	1092	**1.11**	1.08 (0.99, 1.08)	3413	**1.10**	**1.05 (1.02, 1.08)**	5179
1950-1954	1926	1.05	0.94 (0.84, 1.06)	1077	**1.26**	**1.21 (1.08, 1.35)**	3003	**1.12**	1.03 (0.94, 1.12)	4491
1955-1959	1656	0.93	**0.86 (0.75, 0.97)**	1188	**1.43**	**1.37 (1.31, 1.43)**	2844	**1.09**	1.02 (0.94, 1.09)	4364
1960-1964	1300	**0.75**	**0.70 (0.64, 0.77)**	1109	**1.37**	1.31 (1.24, 1.40)	2409	**0.95**	**0.89 (0.83, 0.95)**	4249
1965-1970	1003	**0.68**	**0.64 (0.55, 0.74)**	812	**1.18**	**1.14 (1.08, 1.21)**	1815	**0.84**	**0.79 (0.73, 0.86)**	3611
Highest educational qualification ¥										
None	599	1	1	538	1	1	1137	1	1	3347
O-level	2214	**1.63**	**1.81 (1.54, 2.13)**	1826	**1.50**	**1.36 (1.20, 1.54)**	4040	**1.57**	**1.62 (1.41, 1.86)**	7598
A-level	1570	**1.99**	**2.23 (1.86, 2.68)**	1043	**1.47**	**1.34 (1.18, 1.51)**	2613	**1.75**	**1.81 (1.64, 2.00)**	4403
Higher-level	5077	**2.95**	**3.29 (2.78, 3.88)**	2457	**1.59**	**1.43 (1.32, 1.56)**	7534	**2.30**	**2.38 (2.15, 2.62)**	9629
Accommodation tenure										
Council rental	427	1	1	305	1	1	732	1	1	1873
Private rental	276	1.17	1.00 (0.81, 1.22)	201	**1.20**	1.14 (0.91, 1.41)	477	1.18	1.05 (0.85, 1.29)	1032
Own with mortgage	3447	**1.51**	1.20 (0.99, 1.45)	258	**1.57**	**1.42 (1.15, 1.76)**	6005	**1.53**	**1.28 (1.06, 1.55)**	10025
Own	5310	**1.93**	**1.35 (1.15, 1.60)**	2800	**1.43**	**1.43 (1.15, 1.79)**	810	**1.72**	**1.38 (1.15, 1.65)**	12047
Ethnicity										
White	8616	1	1	5210	1	1	13840	1	1	22078
Mixed ethnicity	112	1.00	1.10 (0.84, 1.43)	70	1.03	1.02 (0.79, 1.31)	182	1.01	1.07 (0.84, 1.35)	288
Asian or Asian British§	251	**0.71**	**0.79 (0.68, 0.91)**	179	**0.84**	**0.84 (0.75, 0.93)**	431	**0.76**	**0.80 (0.73, 0.88)**	909
Black or Black British	279	**0.65**	**0.77 (0.67, 0.89)**	252	0.97	1.00 (0.95, 1.05)	532	**0.77**	**0.86 (0.80, 0.93)**	1097
Chinese	97	**2.00**	**2.05 (1.51, 2.79)**	42	**1.43**	**1.41 (1.21, 1.63)**	139	**1.79**	**1.78 (1.39, 2.27)**	124
Other	104	**0.55**	**0.59 (0.46, 0.77)**	94	0.84	0.84 (0.74, 1.07)	200	**0.66**	**0.70 (0.58, 0.86)**	481

*OR = Odds Ratio. 95% CI = 95% confidence interval. ** Adjusted by year of birth, sex, educational qualification, accommodation tenure, ethnicity, variance adjustment for test centre. ¥O level: State examination at age 16 years; A level: State examination at age 18 years. § Asian category includes Indian, Pakistani and Bangladeshi. **Bold** fonts indicate significant associations at the 5% level.
