# Supplementary material for: Temporal trends in frequency, type and severity of myopia and associations with key environmental risk factors in the UK: Findings from the UK Biobank Study
Source: PLoS One. 2022 Jan 19;17(1):e0260993. doi: 10.1371/journal.pone.0260993 (PMC8769366; doi:10.1371/journal.pone.0260993)
Supplement: S3 Table — *OR = Odds Ratio. 95% CI = 95% confidence interval. ** Adjusted by year of birth, sex, educational qualification, accommodation tenure, ethnicity, variance adjustment for test centre. ¥O level: State examination at age 16 years; A level: State examination at age 18 years. §Asian category includes Indian, Pakistani and Bangladeshi. Bold fonts indicate significant associations at the 5% level. (PDF) [file pone.0260993.s007.pdf]

S2.2_Table: Association of myopia (all, childhood-onset and adult-onset), with socio-demographic and environmental factors in **males.**

	Childhood-onset myopia			Adult-onset myopia			All myopia			Emmetropia
Factors	N 6929	Unadjusted OR* (95% CI)	Adjusted OR ** (95% CI)	N 5821	Unadjusted OR* (95%CI)	Adjusted OR ** (95% CI)	N 12750	Unadjusted OR* (95%CI)	Adjusted OR ** (95% CI)	N 22246
Year of birth	N			N			N			N
1939-1944	1214	**1**	1	850	**1**	**1**	2064	**1**	1	3556
1945-1949	1811	1.09	1.02 (0.92, 1.12)	1300	**1.12**	1.07 (0.98, 1.18)	3111	1.10	1.04 (0.95, 1.14)	4855
1950-1954	1387	1.11	1.03 (0.80, 1.19)	1066	1.21	1.15 (1.07, 1.24)	2453	**1.15**	1.08 (0.99, 1.18)	3673
1955-1959	1016	**0.86**	**0.86 (0.75, 0.99)**	1025	**1.24**	**1.20 (1.15, 1.24)**	2041	1.02	1.00 (0.92, 1.08)	3459
1960-1964	814	**0.68**	**0.72 (0.60, 0.86)**	913	1.09	1.07 (0.95, 1.19)	1727	**0.85**	**0.86 (0.78, 0.95)**	3504
1965-1970	687	**0.63**	**0.66 (0.55, 0.78)**	667	**0.87**	**0.86 (0.82, 0.88)**	1354	**0.73**	**0.73 (0.67, 0.80)**	3199
Highest educational qualification ¥										
None	385	1	1	487	1	1	872	1	1	3391
O-level	1212	**1.78**	**2.00 (1.87, 2.16)**	1443	**1.68**	**1.67 (1.46, 1.88)**	2655	**1.73**	**1.83 (1.67, 2.00)**	5992
A-level	1223	**2.46**	**2.63 (2.35, 2.95)**	1158	**1.84**	**1.81 (1.54, 2.10)**	2381	**2.11**	**2.17 (1.93, 2.44)**	4380
Higher-level	4109	**4.27**	**4.62 (4.06, 5.27)**	2733	**2.24**	**2.22 (1.95, 2.48)**	6842	**3.13**	**3.25 (2.94, 3.59)**	8483
Accommodation tenure										
Council rental	332	1	1	310	1	1	642	1	1	1711
Private rental	288	1.22	0.96 (0.74, 1.24)	241	1.09	0.97 (0.84, 1.12)	529	1.16	0.96 (0.78, 1.17)	1220
Own with mortgage	2441	**1.37**	0.96 (0.76, 1.22)	2444	**1.47**	**1.24 (1.07, 1.43)**	4885	**1.42**	1.09 (0.91, 1.29)	9182
Own	3868	**1.97**	1.18 (0.88, 1.60)	2826	**1.54**	**1.31 (1.12, 1.51)**	6694	**1.76**	1.24 (0.99, 1.55)	10133
Ethnicity										
White	6407	1	1	5201	1	1	11639	1	1	1963
Mixed ethnicity	51	0.81	0.95 (0.73, 1.23)	49	0.96	1.03 (0.83, 1.29)	100	0.88	0.99(0.88, 1.11)	192
Asian or Asian British§	248	**0.71**	**0.76 (0.64, 0.90)**	251	0.88	0.92 (0.80, 1.06)	501	**0.79**	**0.83 (0.73, 0.95)**	1074
Black or Black British	101	**0.34**	**0.41 (0.28, 0.60)**	192	**0.80**	0.89 (0.78, 1.00)	295	**0.55**	**0.64 (0.52, 0.78)**	911
Chinese	69	**3.11**	**2.85 (1.96, 4.15)**	15	0.83	0.85 (0.64, 1.11)	84	**2.09**	**1.95 (1.38, 2.74)**	68
Other	53	**0.47**	**0.50 (0.42, 0.61)**	78	**0.84**	0.92 (0.85, 1.00)	131	**0.64**	**0.69 (0.64, 0.73)**	348

*OR = Odds Ratio. 95% CI = 95% confidence interval. ** Adjusted by year of birth, sex, educational qualification, accommodation tenure, ethnicity, variance adjustment for test centre. ¥O level: State examination at age 16 years; A level: State examination at age 18 years. § Asian category includes Indian, Pakistani and Bangladeshi. **Bold** fonts indicate significant associations at the 5% level.
