# Supplementary material for: Temporal trends in frequency, type and severity of myopia and associations with key environmental risk factors in the UK: Findings from the UK Biobank Study
Source: PLoS One. 2022 Jan 19;17(1):e0260993. doi: 10.1371/journal.pone.0260993 (PMC8769366; doi:10.1371/journal.pone.0260993)
Supplement: S4 Table — ¥O level: State examination at age 16 years; A level: State examination at age 18 years. §Asian category includes Indian, Pakistani and Bangladeshi. (PDF) [file pone.0260993.s008.pdf]

S3_Table: Frequency of myopia (all, childhood-onset and adult-onset), emmetropia and hypermetropia in the UK Biobank population: distribution of socio-demographic and environmental factors.

| **Factor** | **Childhood-onset myopia** | | **Adult-onset myopia** | | **All myopia** | | **Emmetropia** | | **Hypermetropia** | |
|---|---|---|---|---|---|---|---|---|---|---|
| N = 107,442 | n | % (95% CI) | n | % (95% CI) | n | % (95% CI) | n | % (95% CI) | n | % (95% CI) |
| Population % | 16,786 | 15.6 (15.4, 15.8) | 12,066 | 11.2 (11.0,11.4) | 28,852 | 26.9 (26.6, 27.1) | 48,960 | 45.6 (45.3,45.9) | 29,630 | 27.6 (27.3,27.8) |
| Year of birth | | | | | | | | | | |
| 1939-44 | 2,529 | 12.6 (12.2,13.1) | 1,482 | 7.4 (7.0,7.8) | 4,011 | 20.0 (19.5,20.6) | 6,884 | 34.4 (33.7,35.0) | 9,147 | 45.6 (45.0,46.3) |
| 1945-49 | 4,219 | 15.6 (15.2,16.1) | 2,473 | 9.2 (8.8,9.5) | 6,692 | 24.8 (24.3,25.3) | 10,353 | 38.4 (37.8,39.0) | 9,918 | 36.8 (36.2,37.4) |
| 1950-54 | 3,378 | 17.8 (17.2,18.3) | 2,207 | 11.6 (11.2,12.1) | 5,585 | 29.4 (28.8,30.0) | 8,418 | 44.3 (43.6,45.0) | 4,995 | 26.3 (25.7,26.9) |
| 1955-59 | 2,722 | 16.9 (16.3,17.5) | 2,263 | 14.0 (13.5,14.6) | 4,985 | 30.9 (30.2,31.6) | 8,081 | 50.1 (49.3,50.8) | 3,073 | 19.0 (18.4,19.7) |
| 1960-64 | 2,182 | 15.6 (15.0,16.2) | 2,104 | 15.0 (14.4,15.6) | 4,286 | 30.6 (29.8,31.4) | 8,071 | 57.6 (56.8,58.4) | 1,653 | 11.8 (11.3,12.3) |
| 1965-70 | 1,756 | 15.6 (14.9,16.2) | 1,537 | 13.6 (13.0,14.3) | 3,293 | 29.2 (28.3,30 0) | 7,153 | 63.4 (62.5,64.2) | 844 | 7.5 (7.0,8.0) |
| Sex | | | | | | | | | | |
| Female | 9,689 | 16.6 (16.3,16.9) | 6,048 | 10.3 (10.1,10.6) | 15,737 | 26.9 (26.6,27.3) | 25,823 | 44.2 (43.8,44.6) | 16,885 | 28.9 (28.5,29.3) |
| Male | 7,097 | 14.5 (14.2,14.8) | 6,018 | 12.3 (12.0,12.6) | 13,115 | 26.8 (26.4,27.2) | 23,137 | 47.2 (46.8,47.7) | 12,745 | 26.0 (25.6,26.4) |
| Highest educational qualification¥ | | | | | | | | | | |
| None | 1,009 | 6.4 (6.0,6.8) | 1,054 | 6.7 (6.3,7.1) | 2,063 | 13.1 (12.6,13.6) | 6,951 | 44.1 (43.3,44.8) | 6762 | 42.9 (42.1,43.6) |
| O-level | 3,500 | 12.4 (12.0,12.8) | 3,344 | 11.8 (11.5,12 2) | 6,844 | 24.2 (23.7,24.7) | 13,849 | 49.0 (48.4,49.6) | 7,579 | 26.8 (26.3,27.3) |
| A-level | 2,854 | 14.9 (14.4,15.4) | 2,248 | 11.7 (11.3,12.2) | 5,102 | 26.6 (26.0,27.3) | 8,974 | 46.8 (46.1,47.5) | 5,085 | 26.5 (25.9,27.2) |
| Higher-level | 9,329 | 21.8 (21.4,22.2) | 5,292 | 12.4 (12.1,12.7) | 14,621 | 34.2 (33.7,34.6) | 18,446 | 43.1 (42.7,43.6) | 9,700 | 22.7 (22.3,23.1) |
| *Missing* | 94 | - | 128 | - | 222 | - | 740 | - | 504 | - |
| Accommodation tenure | | | | | | | | | | |

| | | | | | | | | | | |
|---|---|---|---|---|---|---|---|---|---|---|
| Rent from council | 782 | 10.7 (10.0,11.4) | 642 | 8.8 (8.2,9.5) | 1,424 | 19.5 (18.6,20.4) | 3,720 | 51.0 (49.8,52.1) | 2,155 | 29.5 (28.5,30.6) |
| Rent from private | 571 | 13.1 (12.1,14.2) | 455 | 10.5 (9.6,11.4) | 1,026 | 23.6 (22.3,24.9) | 2,328 | 53.5 (52.0,55.0) | 997 | 22.9 (21.7,24.2) |
| Own with mortgage | 5,937 | 15.7 (15.3,16.1) | 5,044 | 13.3 (13.0,13.7) | 10,981 | 29.0 (28.6,29.5) | 19,441 | 51.4 (50.9,51.9) | 7,431 | 19.6 (19.2,20.0) |
| Own outright | 9,251 | 16.6 (16.3,16.9) | 5,684 | 10.2 (9.9,10.4) | 14,935 | 26.4 (26.4,27.1) | 22,458 | 40.2 (39.8,40.6) | 18,444 | 33.0 (32.6,33.4) |
| *Missing* | 245 | - | 241 | - | 486 | - | 1,013 | - | 603 | - |
| Ethnicity | | | | | | | | | | |
| White | 15,257 | 15.9 (15.7,16.2) | 10,659 | 11.1 (10.9,11.3) | 25,916 | 27.6 (26.8,27.3) | 42,625 | 44.5 (44.2,44.8) | 27,242 | 28.4 (28.2,28.7) |
| Mixed ethnicity | 168 | 17.2 (15.0,19.8) | 122 | 12.5 (10.6,14.8) | 290 | 29.8 (27.0,32.7) | 516 | 53.0 (49.8,56.1) | 168 | 17.2 (15.0,19.8) |
| Asian or Asian British§ | 528 | 13.1 (12.1,14.2) | 472 | 11.7 (10.7,12.7) | 1,000 | 24.8 (23.5,26.2) | 2,185 | 54.2 (52.6,55.7) | 847 | 21.0 (18.5,21.0) |
| Black or Black British | 396 | 10.5 (9.6,11.6) | 479 | 12.8 (11.7,13.9) | 875 | 23.3 (22.0,24.7) | 2,143 | 57.0 (55.4,58.6) | 740 | 19.7 (18.5,21.0) |
| Chinese | 169 | 34.5 (30.3,38.8) | 63 | 12.7 (10.1,16.1) | 232 | 47.4 (42.9,51.8) | 205 | 41.8 (37.5,46.3) | 53 | 10.8 (8.3,13.9) |
| Other | 166 | 10.2 (8.8,11.8) | 189 | 11.6 (10.2,13.3) | 355 | 21.8 (19.9,23.9) | 908 | 55.8 (53.4,58.2) | 364 | 22.4 (20.4,24.5) |
| *Missing* | 102 | - | 82 | - | 184 | - | 378 | - | 216 | - |

¥O level: State examination at age 16 years; A level: State examination at age 18 years. § Asian category includes Indian, Pakistani and Bangladeshi.
