# Supplementary material for: Temporal trends in frequency, type and severity of myopia and associations with key environmental risk factors in the UK: Findings from the UK Biobank Study
Source: PLoS One. 2022 Jan 19;17(1):e0260993. doi: 10.1371/journal.pone.0260993 (PMC8769366; doi:10.1371/journal.pone.0260993)
Supplement: S5 Table — ¥O level: State examination at age 16 years; A level: State examination at age 18 years. §Asian category includes Indian, Pakistani and Bangladeshi. (PDF) [file pone.0260993.s009.pdf]

S4_Tables: Frequency of myopia (all, childhood-onset and adult-onset), emmetropia and hypermetropia: distribution of socio-demographic and environmental factors, by year of birth (1939-44, 1945-49, 1950-54, 1955-59, 1960-64 and 1965-69).

**Factor**	**Childhood-onset myopia**		**Adult-onset myopia**		**All myopia**		**Emmetropia**		**Hypermetropia**	
**Year of Birth**	n	% (95% CI)	n	% (95% CI)	n	% (95% CI)	n	% (95% CI)	n	% (95% CI)
**1939 – 44 N=20,042**	2529	12.6 (12.2, 13.1)	1482	7.4 (7.0, 7.8)	4011	20.0 (19.5, 20.6)	6884	34.4 (32.7, 35.0)	9147	45.6 (45.0, 46.3)
**Sex**										
Female	1284	12.8 (12.2, 13.5)	605	6.0 (5.6, 13.5)	1889	18.9 (18.1, 19.6)	3198	31.9 (31.0, 32.8)	4929	49.2 (48.2, 50.2)
Male	1245	12.4 (11.8, 13.1)	877	8.8 (8.2, 9.3)	2122	21.2 (20.4, 22.0)	3686	36.8 (35.8, 37.7)	4218	42.1 (41.1, 43.0)
**Highest educational qualification¥**										
None	382	6.7 (6.1, 7.4)	288	5.1 (4.5, 5.6)	670	11.7 (10.9, 12.6)	2022	35.4 (34.2, 36.7)	3014	52.8 51.5, 54.1)
O-level	558	12.5 (11.6, 13.5)	367	8.2 (7.4, 9.1)	925	20.7 (19.5, 21.9)	1475	33.0 (31.7, 34.4)	2066	46.3 (44.8, 47.7)
A-level	383	12.7 (11.6, 14.0)	250	8.3 (7.4, 9.4)	633	21.1 (19.6, 22.6)	1073	35.7 (34.0, 37.4)	1300	43.3 (41.5, 45.0)
Higher-level	1193	18.2 (17.3, 19.2)	562	8.6 (7.9, 9.3)	1755	26.8 (25.7, 27.9)	2200	33.6 (32.4, 34.7)	2598	39.7 (38.5, 40.8)
Missing	13	-	15	-	28	-	114	-	169	-
**Accommodation tenure**										
Rent from council	98	9.4 (7.8, 11.3)	70	6.7 (5.3, 8.4)	168	16.1 (14.0, 18.5)	353	33.9 (31.0, 36.8)	521	50.0 (47.1, 53.0)
Rent from private	30	7.8 (5.5, 10.9)	19	4.9 (3.9, 7.6)	49	12.4 (9.7, 16.4)	140	36.2 (31.5, 41.1)	198	51.2 (46.2, 56.1)
Own with mortgage	214	11.7 (10.3, 13.3)	148	8.1 (6.9, 9.5)	362	19.9 (18.1, 21.7)	624	34.2 (32.1, 36.4)	838	45.9 (43.7, 48.2)
Own outright	2156	13.2 (12.6, 13.7)	1213	7.4 (7.0, 7.8)	3369	20.6 (19.9, 21.1)	5625	34.4 (33.6, 35.1)	7392	45.1 (44.1, 45.8)
Missing	31	-	32	-	63	-	132	-	198	-
**Ethnicity**										
White	2432	12.9	1387	7.4	3819	20.2	6410	34.0	8638	45.8

		(12.4, 13.4)		(7.0, 7.7)		(19.7, 20.8)		(33.8, 34.7)		(45.1, 46.5)
Mixed ethnicity	6	6.7<br>(3.0, 13.3)	7	7.9<br>(3.7, 15.8)	13	14.6<br>(8.6, 23.8)	38	42.7<br>(32.7, 53.3)	38	42.9<br>(32.7, 53.3)
Asian or Asian British§	38	8.7<br>(6.4, 11.7)	35	8.0<br>(5.8, 11.0)	73	10.7<br>(13.5, 20.5)	185	42.3<br>(37.8, 47.0)	179	41.0<br>(36.5, 45.7)
Black or Black British	10	3.1<br>(1.7, 5.7)	27	8.5<br>(5.9, 12.1)	37	11.6<br>(8.5, 15.6)	128	40.1<br>(34.9, 45.6)	154	48.3<br>(42.8, 53.8)
Chinese	13	27.1<br>(16.1, 41.8)	3	6.3<br>(2.0, 18.0)	16	33.3<br>(21.2, 48.2)	19	39.6<br>(26.4, 54.4)	13	27.1<br>(16.1, 41.8)
Other	12	8.2<br>(4.7, 13.9)	16	10.9<br>(6.7, 17.1)	28	19.1<br>(13.4, 26.3)	58	39.5<br>(31.8, 47.7)	61	41.5<br>(33.7, 48.7)
Missing	18	-	7	-	25	-	46	-	64	-

¥O level: State examination at age 16 years; A level: State examination at age 18 years. §Asian category includes Indian, Pakistani and Bangladeshi

Factor	Childhood-onset myopia		Adult-onset myopia		All myopia		Emmetropia		Hypermetropia	
**Year of Birth**	n	% (95% CI)	n	% (95% CI)	n	% (95% CI)	n	% (95% CI)	n	% (95% CI)
**1944-49 N=26,963**	4219	15.6 (15.2, 16.1)	2473	9.2 (8.8, 935)	6692	24.8 (24.3, 25.3)	10,353	33.4 (37.8, 39.0)	9918	36.8 (36.2, 37.4)
**Sex**										
Female	2373	16.3 (15.7, 16.9)	1137	7.8 (9.4, 8.3)	3510	24.1 (23.4, 24.8)	5339	36.7 (35.9, 37.5)	5704	39.2 (38.4, 40.0)
Male	1846	14.9 (14.3, 15.5)	1336	10.8 (10.2, 11.3)	3182	25.6 (24.9, 26.4)	5014	40.4 (39.5, 41.2)	4214	34.0 (33.1, 34.8)
**Highest educational qualification¥**										
None	353	6.8 (6.1, 7.5)	344	6.6 (5.9, 7.3)	697	13.4 (12.5, 14.3)	2136	40.9 (39.6, 42.3)	2387	45.7 (44.4, 47.1)
O-level	901	14.2 (13.3, 15.1)	633	10.1 (9.2, 10.7)	1534	24.1 (23.1, 25.2)	2491	39.2 (38.0, 40.4)	2332	36.7 (35.5, 37.9)
A-level	719	15.1 (14.1, 16.1)	450	9.4 (8.6, 10.3)	1169	24.5 (23.3, 25.3)	1845	38.7 (37.3, 40.0)	1756	36.8 (35.5, 38.2)
Higher-level	2217	21.6 (20.8, 22.4)	1014	9.9 (9.3, 10.4)	3231	31.4 (30.5, 32.3)	3753	36.5 (35.6, 37.4)	3303	32.1 (31.2, 33.0)
Missing	29	-	32	-	61	-	128	-	140	-
**Accommodation tenure**										
Rent from council	164	11.5 (10.0, 13.3)	95	6.7 (5.5, 8.1)	259	18.2 (16.3, 20.3)	535	37.7 (35.2, 40.2)	629	44.1 (41.6, 46.7)
Rent from private	83	13.5 (11.0, 16.5)	51	8.3 (6.4, 10.8)	134	21.8 (18.7, 25.3)	229	37.3 (33.5, 41.2)	251	40.9 (37.0, 44.8)
Own with mortgage	721	14.6 (13.7, 15.6)	484	9.8 (9.0, 10.7)	1205	24.4 (23.3, 25.7)	1939	39.3 (38.0, 40.7)	1787	36.2 (34.9, 37.6)
Own outright	3205	16.4 (15.9, 16.9)	1798	9.2 (8.8, 9.6)	5003	23.6 (25.0, 26.2)	7471	38.2 (37.5, 38.9)	7083	36.2 (35.5, 36.9)
Missing	46	-	45	-	91	-	179	-	170	-
**Ethnicity**										
White	4042	16.0 (15.5, 16.4)	2294	9.1 (8.9, 9.4)	6336	25.0 (25.5, 25.6)	9681	38.2 (37.6, 38.8)	9313	36.8 (36.2, 37.4)
Mixed ethnicity	24	20.9 (14.3, 29.3)	11	9.6 (5.3, 16.6)	35	30.4 (22.6, 39.6)	40	34.8 (26.5, 44.1)	40	34.8 (26.5, 44.1)

Asian or Asian British§	71	11.4<br>(9.1, 14.2)	75	12.1<br>(9.7, 14.9)	146	23.5<br>(20.3, 27.0)	258	41.5<br>(37.7, 45.4)	218	35.1<br>(31.4, 38.9)
Black or Black British	22	5.5<br>(3.6, 8.2)	42	10.4<br>(7.8, 13.8)	64	15.8<br>(12.6, 19.8)	169	41.9<br>(37.2, 46.8)	170	42.2<br>(37.4, 47.0)
Chinese	19	25.3<br>(16.6, 36.6)	8	10.7<br>(5.3, 20.2)	27	36.0<br>(25.8, 47.9)	31	41.3<br>(30.6, 53.0)	17	22.7<br>(14.4, 33.8)
Other	21	8.8<br>(5.8, 13.1)	24	10.0<br>(6.8, 14.6)	45	18.8<br>(14.3, 24.3)	98	41.0<br>(34.9, 47.4)	96	40.2<br>(34.1, 46.6)
Missing	20	-	19	-	39	-	76	-	64	-

¥O level: State examination at age 16 years; A level: State examination at age 18 years. §Asian category includes Indian, Pakistani and Bangladeshi

Factor	Childhood-onset myopia		Adult-onset myopia		All myopia		Emmetropia		Hypermetropia	
**Year of Birth**	n	% (95% CI)	n	% (95% CI)	n	% (95% CI)	n	% (95% CI)	n	% (95% CI)
**1950-54 N=18,998**	3378	17.8 (17.2, 18.3)	2207	11.6 (11.2, 12.1)	5585	29 (28.8, 30.0)	8418	44.3 (43.6, 45.0)	4915	26.3 (25.7, 27.0)
**Sex**										
Female	1966	18.5 (17.7, 19.2)	1107	10.4 (9.8, 11.0)	3073	28.8 (28.0, 29.9)	4630	43.5 (42.5, 44.4)	2951	27.7 (26.9, 28.6)
Male	1412	16.9 (16.4, 17.7)	1100	13.2 (12.5, 13.9)	2512	30.1 (29.1, 31.1)	3788	45.4 (44.3, 46.5)	2044	24.5 (23.6, 25.4)
**Highest educational qualification¥**										
None	137	6.1 (5.2, 7.2)	188	8.4 (7.3, 9.6)	325	14.5 (13.1, 16.0)	1123	50.0 (47.9, 52.0)	800	35.6 (33.6, 37.6)
O-level	643	13.6 (12.6, 14.5)	575	12.1 (11.2, 13.1)	1218	25.7 (24.4, 24.9)	2226	46.9 (45.5, 48.3)	1303	27.5 (26.2, 28.7)
A-level	578	16.1 (14.9, 17.3)	413	11.5 (10.5, 12.6)	991	27.6 (26.2, 29.1)	1632	27.5 (43.8, 47.1)	969	27.0 (25.5, 28.5)
Higher-level	2003	24.4 (23.6, 25.4)	1005	12.3 11.6, 13.0)	3008	36.8 (35.9, 37.8)	3330	40.7 (39.7, 41.8)	1840	22.5 (21.6, 23.4)
Missing	17	-	26	-	43	-	107	-	83	-
**Accommodation tenure**										
Rent from council	149	11.9 (10.2, 13.8)	114	9.1 (7.6, 10.8)	263	21.0 (18.8, 23.3)	579	46.2 (43.5, 49.0)	411	32.8 (30.3, 35.5)
Rent from private	81	13.5 (10.9, 16.4)	61	10.1 (98.0, 12.8)	142	23.6 (20.4, 27.2)	289	48.0 (44.0, 52.0)	171	28.4 (24.9, 32.2)
Own with mortgage	1100	16.6 (15.8, 17.5)	801	12.1 (11.3, 12.9)	1901	28.7 (27.7, 29.8)	2993	45.3 (44.0, 46.5)	1720	26.0 (25.0, 27.1)
Own outright	2006	19.7 (18.9, 30.4)	1194	11.7 (11.1, 12.3)	3200	31.4 (30.5, 32.3)	4408	43.2 (42.2, 44.1)	2599	25.5 (24.6, 26.3)
Missing	42	-	37	-	79	-	149	-	94	-
**Ethnicity**										
White	3123	18.3 (17.7, 18.9)	1985	11.6 (11.1, 12.1)	5108	29.9 (29.2, 30.6)	7522	44.0 (43.3, 44.8)	4454	26.1 (25.4, 26.7)
Mixed ethnicity	29	20.7 (14.7, 28.3)	15	10.7 (6.5, 17.1)	44	31.4 (24.2, 39.7)	64	45.7 (37.6, 54.1)	32	22.9 (16.6, 30.6)

Asian or Asian British§	86	12.0 (9.8, 14.6)	85	11.8 (9.7, 14.4)	171	23.8 (20.8, 27.0)	324	45.1 (41.4, 48.7)	224	31.2 (27.9, 34.6)
Black or Black British	40	7.4 (5.5, 10.0)	62	11.5 (9.1, 14.5)	102	19.0 (15.8, 22.5)	279	52.0 (47.7, 56.2)	156	29.1 (25.4, 33.0)
Chinese	49	43.7 (34.7, 53.2)	11	9.8 (5.5, 17.0)	60	53.6 (44.2, 62.7)	41	36.6 (28.1, 46.0)	11	9.8 (5.5, 17.0)
Other	33	11.5 (8.3, 15.8)	37	12.9 (9.5, 17.4)	70	24.5 (19.8, 29.8)	132	46.2 (40.4, 52.0)	84	29.4 (24.4, 34.9)
Missing	18	-	12	-	30	-	56	-	34	-

¥O level: State examination at age 16 years; A level: State examination at age 18 years. §Asian category includes Indian, Pakistani and Bangladeshi

Factor	Childhood-onset myopia		Adult-onset myopia		All myopia		Emmetropia		Hypermetropia	
**Year of Birth**	n	% (95% CI)	n	% (95% CI)	n	% (95% CI)	n	% (95% CI)	n	% (95% CI)
**1955-59 N=16,139**	2722	16.9 (16.3, 17.5)	2263	14.0 (13.5, 14.6)	4985	30.9 (30.2, 31.6)	8081	90.1 (49.3, 50.8)	3073	19.0 (18.4, 19.7)
**Sex**										
Female	1683	18.2 (17.4, 19.0)	1213	15.1 (12.4, 13.8)	2896	31.3 (30.4, 32.3)	4500	48.7 (47.7, 49.7)	1848	20 (19.2, 20.8)
Male	1039	13.1 (14.2, 15.9)	1050	15.2 (14.4, 16.1)	2089	30.3 (29.2, 31.4)	3581	51.9 (50.8, 53.1)	1225	17.8 (16.9, 18.7)
**Highest educational qualification¥**										
None	75	5.7 (4.5, 7.1)	121	9.2 (7.7, 10.8)	196	14.8 (13.0, 16.8)	757	57.3 (54.6, 60.0)	369	27.9 (25.6, 30.4)
O-level	559	11.8 (10.9, 12.8)	704	14.9 (13.9, 15.9)	1263	26.7 (25.5, 28.0)	2487	52.6 (51.1, 54.0)	981	20.7 (19.6, 21.9)
A-level	495	15.7 (14.5, 17.1)	459	14.6 (13.4, 15.8)	954	30.3 (28.7, 31.9)	1619	51.4 (50.0, 53.2)	576	18.3 (17.0, 20.0)
Higher-level	1583	23.4 (22.4, 24.4)	968	14.3 (13.5, 15.2)	2551	37.7 (36.6, 38.9)	3116	46.1 (44.9, 47.3)	1092	16.2 (15.3, 17.1)
Missing	10	-	11	-	21	-	102	-	55	-
**Accommodation tenure**										
Rent from council	121	9.6 (8.1, 11.4)	134	10.7 (9.1, 12.5)	255	20.3 (18.2, 32.6)	691	55.1 (52.3, 57.8)	309	24.6 (22.3, 27.1)
Rent from private	107	14.0 (11.7, 16.6)	96	12.6 (10.4, 15.1)	203	26.5 (23.5, 29.8)	395	51.6 (48.4, 55.2)	167	21.8 (19.0, 24.9)
Own with mortgage	1326	15.8 (15.0, 16.6)	1235	14.7 (14.0, 15.5)	2561	30.5 (29.5, 31.5)	4259	50.7 (49.7, 51.8)	1575	18.8 (17.9, 19.6)
Own outright	1133	20.9 (19.8, 22.0)	767	14.1 (13.2, 15.1)	1900	35.0 (34.7, 36.3)	257	47.5 (46.1, 48.8)	956	17.6 (16.6, 18.6)
Missing	35	-	31	-	66	-	159	-	68	-
**Ethnicity**										
White	2393	17.2 (16.6, 17.8)	1990	14.3 (13.7, 14.9)	4383	31.5 (30.7, 32.2)	6881	49.4 (48.6, 50.2)	2662	19.1 (18.5, 19.8)
Mixed ethnicity	36	19.2 (14.1, 25.5)	19	10.1 (6.5, 15.4)	55	29.3 (23.1, 36.2)	103	54.8 (47.6, 61.8)	3	16.0 (11.4, 22.0)

Asian or Asian British§	119	16.5 (13.9, 19.4)	81	11.2 (9.1, 13.7)	200	27.7 (24.5, 31.0)	393	54.4 (50.7, 58.0)	130	18.0 (15.3, 21.0)
Black or Black British	99	12.3 (10.2, 14.8)	113	14.0 (11.8, 16.6)	212	26.3 (23.4, 29.5)	442	54.9 (51.4, 58.3)	151	18.8 (16.2, 21.6)
nese	25	27.8 (19.4, 38.1)	15	16.7 (10.2, 26.0)	40	44.4 (34.4, 55.0)	43	47.8 (37.5, 58.2)	7	7.8 (3.7, 15.6)
Other	38	12.8 (9.4, 17.1)	30	10.1 (7.1, 14.1)	68	22.8 (18.4, 28.0)	165	55.4 (49.6, 60.1)	65	21.8 (17.5, 26.9)
Missing	12	-	15	-	27	-	54	-	28	-

¥O level: State examination at age 16 years; A level: State examination at age 18 years. §Asian category includes Indian, Pakistani and Bangladeshi

Factor	Childhood-onset myopia		Adult-onset myopia		All myopia		Emmetropia		Hypermetrop	
**Year of Birth**	n	% (95% CI)	n	% (95% CI)	n	% (95% CI)	n	% (95% CI)	n	% (95% CI)
**1960-64 N=14,010**	2182	15.6 (15.0, 16.2)	2104	15.0 (14.4, 15.6)	4286	30.6 (29.8, 31.4)	8071	57.6 (56.8, 58.4)	1653	11.8 (11.3, 12.3)
**Sex**										
Female	1336	17.0 (16.2, 17.8)	1145	14.5 (13.8, 15.3)	2481	31.5 (30.5, 32.5)	4395	55.8 (54.7, 56.9)	997	12.7 (11.9, 13.4)
Male	846	13.8 (12.9, 14.7)	959	15.6 (14.7, 16.6)	1805	29.4 (28.3, 30.6)	3676	59.9 (58.7, 61.1)	656	10.7 (9.9, 11.5)
**Highest educational qualification¥**										
None	44	5.7 (4.3, 7.6)	71	9.2 (7.4, 11.5)	115	14.9 (12.6, 17.6)	520	67.5 (64.1, 70.8)	135	17.5 (15.0, 20.4)
O-level	485	10.6 (9.8, 11.5)	641	14.0 (13.0, 15.1)	112	24.6 (23.4, 25.9)	2846	62.3 (60.8, 63.7)	2578	13.1 (12.1, 14.1)
A-level	393	15.1 (13.7, 16.5)	399	15.3 (14.0, 16.7)	792	30.3 (28.6, 32.1)	1502	57.5 (55.6, 59.4)	317	12.1 (10.9, 13.5)
Higher-level	1250	21.3 (20.3, 22.4)	968	16.5 (15.6, 17.5)	2218	37.9 (36.6, 39.1)	3075	52.5 (51.2, 53.8)	565	9.6 (8.9, 10.4)
Missing	10	-	25	-	35	-	128	-	38	-
**Accommodation tenure**										
Rent from council	131	10.2 (8.7, 12.0)	132	10.3 (8.7, 12.1)	263	20.5 (18.4, 22.8)	831	64.7 (62.1, 67.3)	190	14.8 (13.0, 16.8)
Rent from private	103	11.9 (9.9, 14.2)	108	12.4 (10.4, 14.8)	211	24.3 (21.5, 27.2)	534	61.5 (58.2, 64.6)	124	14.3 (12.1, 16.8)
Own with mortgage	1418	16.3 (15.5, 17.1)	1333	15.3 (14.6, 16.1)	2751	31.6 (30.7, 32.6)	4971	57.2 (56.1, 58.2)	974	11.2 (10.6, 11.9)
Own outright	480	17.0 (16.7, 18.5)	472	16.8 (15.4, 18.2)	952	33.8 (32.1, 35.6)	1552	55.1 (53.3, 56.9)	313	11.1 (10.0, 12.3)
Missing	50	-	59	-	109	-	183	-	52	-
**Ethnicity**										
White	1827	15.8 (15.1, 16.5)	1760	15.2 (14.6, 15.9)	3587	31.0 (30.1, 31.8)	6568	56.7 (55.8, 57.6)	1423	12.3 (11.7, 12.9)
Mixed ethnicity	38	15.3 (11.3, 20.4)	39	15.7 (11.7, 20.8)	77	31.1 (25.6, 37.1)	149	60.1 (53.8, 66.0)	22	8.9 (5.9, 13.1)

Asian or Asian British§	99	14.0 (11.6, 16.7)	95	13.4 (11.8, 16.3)	194	27.4 (24.2, 30.8)	446	62.9 (59.3, 66.4)	69	9.7 (7.8, 12.1)
Black or Black British	136	14.7 (12.5, 17.1)	129	13.9 (11.8, 16.3)	265	28.6 (25.8, 31.0)	585	63.2 (60.0, 66.2)	76	8.2 (6.6, 10.2)
Chinese	36	39.1 (29.6, 50.0)	20	21.7 (14.4, 31.5)	56	60.9 (50.4, 70.2)	32	34.8 (25.6, 45.2)	4	4.4 1.6, 11.2)
Other	29	8.8 (6.2, 12.4)	42	12.8 (9.6, 16.8)	71	21.6 (17.4, 26.4)	215	65.4 (60.0, 70.3)	43	13.1 (9.8, 17.2)
Missing	17	-	19	-	36	-	76	-	16	-

¥O level: State examination at age 16 years; A level: State examination at age 18 years. §Asian category includes Indian, Pakistani and Bangladeshi

Factor	Childhood-onset myopia		Adult-onset myopia		All myopia		Emmetropia		Hypermetrop	
**Year of Birth**	n	% (95% CI)	n	% (95% CI)	n	% (95% CI)	n	% (95% CI)	n	% (95% CI)
**1965-69 N=11,290**	1756	15.6 (14.9, 16.2)	1537	13.6 (13.0, 14.3)	3293	29.2 (28.3, 30.0)	7153	63.4 (62.5, 64.2)	844	7.5 (7.0, 8.0)
**Sex**										
Female	1047	17.2 (16.2, 18.1)	841	3.8 (12.9, 14.7)	1888	30.9 (29.8, 32.1)	3761	61.6 (60.4, 62.8)	456	7.5 (6.8, 8.2)
Male	709	13.9 (13.8, 14.6)	696	13.4 (12.5, 14.4)	1405	27.1 (25.9, 28.3)	3392	65.4 (64.1, 66.7)	388	7.5 (6.8, 8.2)
**Highest educational qualification¥**										
None	18	3.5 (2.2, 5.5)	42	8.2 (6.1, 11.0)	60	11.8 (9.2, 14.9)	393	77.1 (73.2, 80.5)	57	11.2 (8.9, 14.2)
O-level	354	10.4 (9.4, 11.5)	424	12.5 (11.4, 13.6)	778	22.9 (21.5, 24.3)	2324	68.3 (66.7, 69.9)	299	8.8 (7.9, 9.8)
A-level	286	14.1 (12.6, 15.6)	277	13.6 (12.2, 15.2)	563	27.7 (25.8, 29.7)	1303	64.1 (62.0, 66.2)	167	8.2 (7.1, 9.5)
Higher-level	1083	21.1 (20.0, 22.2)	775	15.1 (14.1, 16.1)	1858	36.2 (34.9, 37.5)	2972	57.9 (56.6, 59.3)	302	5.9 (5.3, 6.6)
Missing	15	-	19	-	34	-	161	-	19	-
**Accommodation tenure**										
Rent from council	119	11.4 (9.6, 13.5)	97	9.3 (7.7, 11.2)	216	20.7 (18.3, 23.3)	731	70.0 (67.2, 72.7)	97	9.3 (7.7, 8.2)
Rent from private	167	15.0 (13.0, 17.2)	120	10.8 (9.1, 12.7)	287	25.8 (23.3, 28.4)	741	66.5 (63.7, 69.2)	86	7.7 (6.3, 9.4)
Own with mortgage	1158	15.7 (14.9, 13.5)	1043	14.1 (13.3, 14.9)	2201	29.8 (28.7, 30.8)	4655	63.0 (61.9, 64.1)	537	7.3 (6.7, 7.9)
Own outright	271	19.0 (17.0, 21.1)	240	16.8 (14.9, 18.8)	511	35.8 (33.3, 38.3)	815	57.0 (54.4, 59.6)	103	7.2 (6.0, 8.7)
Missing	41	-	37	-	78	-	211	-	21	-
**Ethnicity**										
White	1440	16.0 (15.3, 16.8)	1243	13.8 (13.1, 14.5)	2683	29.8 (28.9, 30.8)	5563	61.8 (60.8, 62.8)	752	8.4 (7.8, 6.8)
Mixed ethnicity	35	18.0 (13.8, 24.2)	31	16.0 (11.4, 21.9)	66	34.0 (27.6, 41.0)	122	62.9 (55.8, 69.4)	6	3.1 (1.4, 6.8)

Asian or Asian British§	115	14.0 (11.8, 16.5)	101	12.3 (10.2, 14.7)	216	26.3 (23.4, 29.4)	579	70.4 (67.2, 73.5)	27	3.3 (2.3, 4.8)
Black or Black British	89	11.6 (9.5, 14.1)	106	13.8 (11.5, 16.4)	195	25.4 (22.4, 28.6)	540	70.3 (67.0, 73.4)	33	4.3 (3.1, 6.0)
Chinese	27	37.0 (26.5, 48.8)	6	8.2 (3.7, 17.4)	33	45.2 (34.5, 69.0)	39	53.4 (41.7, 64.8)	1	1.4
Other	33	10.1 (7.2, 13.8)	40	12.2 (9.1, 16.2)	73	22.3 (18.1, 27.1)	240	73.2 (68.1, 77.7)	15	4.6 (2.8, 7.5)
Missing	17	-	10	-	27	-	70	-	10	-

¥O level: State examination at age 16 years; A level: State examination at age 18 years. §Asian category includes Indian, Pakistani and Bangladeshi
